# Dynamic, state-dependent characteristics of cognitive fluctuations in Lewy body dementia: a magnetoencephalography study

**DOI:** 10.1093/braincomms/fcag236

**Published:** 2026-06-24

**Authors:** Hojjatollah Sadeqi, Babak Ahmadi, Zohreh Morshedizad, Rachael Burke, Bhavana Patel, Nikolaus R McFarland, Melissa J Armstrong, Abbas Babajani-Feremi

**Affiliations:** Department of Neurology, University of Florida, Gainesville, FL 32611, USA; The Norman Fixel Institute of Neurological Diseases, University of Florida Health, Gainesville, FL 32608, USA; Department of Neurology, University of Florida, Gainesville, FL 32611, USA; The Norman Fixel Institute of Neurological Diseases, University of Florida Health, Gainesville, FL 32608, USA; Department of Industrial and Systems Engineering, University of Florida, Gainesville, FL 32611, USA; Department of Neurology, University of Florida, Gainesville, FL 32611, USA; The Norman Fixel Institute of Neurological Diseases, University of Florida Health, Gainesville, FL 32608, USA; Department of Neurology, University of Florida, Gainesville, FL 32611, USA; The Norman Fixel Institute of Neurological Diseases, University of Florida Health, Gainesville, FL 32608, USA; Department of Neurology, University of Florida, Gainesville, FL 32611, USA; The Norman Fixel Institute of Neurological Diseases, University of Florida Health, Gainesville, FL 32608, USA; Department of Neurology, University of Florida, Gainesville, FL 32611, USA; The Norman Fixel Institute of Neurological Diseases, University of Florida Health, Gainesville, FL 32608, USA; Department of Neurology, University of Florida, Gainesville, FL 32611, USA; The Norman Fixel Institute of Neurological Diseases, University of Florida Health, Gainesville, FL 32608, USA; Department of Neurology, University of Florida, Gainesville, FL 32611, USA; The Norman Fixel Institute of Neurological Diseases, University of Florida Health, Gainesville, FL 32608, USA

**Keywords:** cognitive fluctuations (CF), dynamic functional connectivity, hidden Markov modelling (HMM), magnetoencephalography (MEG), Lewy body dementia (LBD)

## Abstract

Cognitive fluctuations are a hallmark clinical feature of Lewy body dementia (LBD), yet their underlying neural mechanisms remain poorly understood. This study aimed to identify dynamic, state-dependent neural signatures of cognitive fluctuations in LBD using magnetoencephalography and dynamic functional connectivity based on hidden Markov modelling. Resting-state magnetoencephalography data were acquired from individuals with LBD, Parkinson’s disease without dementia and cognitively normal controls. Hidden Markov modelling was used to identify transient brain states followed by spectral analyses across regions and states. Additionally, associations between regional spectral power and cognitive fluctuations severity, measured by the Clinician Assessment of Fluctuation, were assessed. Patients with LBD exhibited a distinct pattern of brain dynamics, particularly in two states (States 2 and 6), characterized by increased fractional occupancy of State 2 and markedly reduced occupancy of State 6, contrasting with the more distributed state engagement observed in Parkinson’s disease and normal controls. Spectral analyses revealed widespread slowing in LBD, with elevated theta/beta power ratios in frontal, parietal and visual cortices—most pronounced in States 2 and 6. Region-specific theta/beta power ratio elevations were identified in the anterior cingulate, medial prefrontal cortex, posterior cingulate, dorsal visual stream and auditory cortex. Critically, Clinician Assessment of Fluctuation scores correlated positively with spectral power in low frequency (δ and θ) and negatively with power in the high frequency (α and β), particularly in the ventral visual stream, default mode network hubs and sensorimotor regions. These findings reveal dynamic and spatially specific electrophysiological abnormalities in LBD closely linked to cognitive fluctuations severity, suggesting that magnetoencephalography-hidden-Markov-model characteristics hold promise as biomarkers for diagnosis, monitoring and therapeutic targeting in LBD.

## Teaser

Sadeqi *et al*. showed that Lewy body dementia is characterized by abnormal, state dependent brain dynamics measured with a hidden Markov model of magnetoencephalography data, including altered brain state occupancy and pronounced low frequency spectral slowing, which strongly correlated with the clinical severity of cognitive fluctuations.

## Graphical Abstract

**Graphical Abstract:**
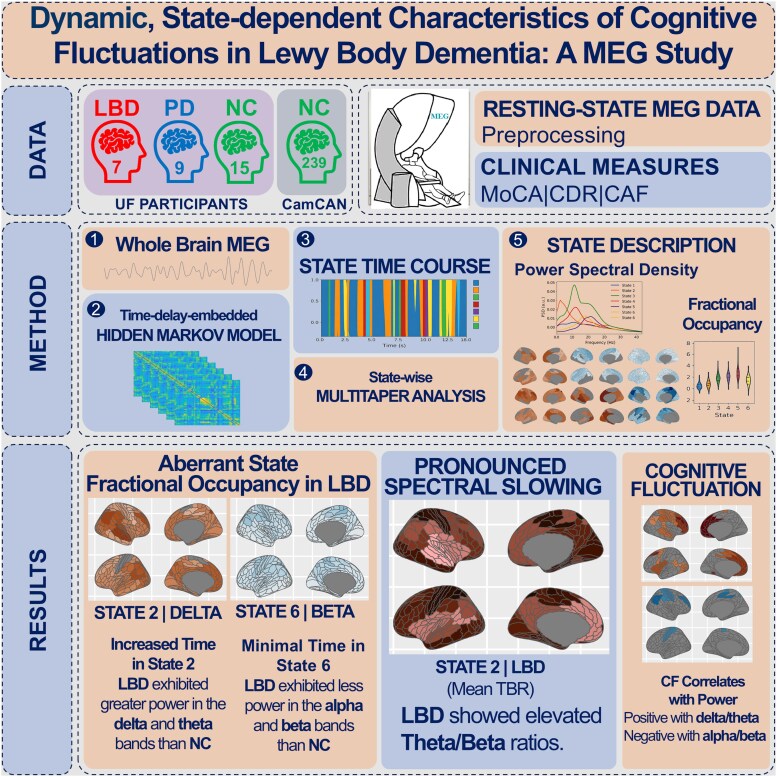

## Introduction

Lewy body dementia (LBD), an umbrella term encompassing dementia with Lewy bodies (DLB) and Parkinson’s disease dementia (PDD),^1^ is the second most common neurodegenerative dementia after Alzheimer’s disease (AD),^2,3^ accounting for ∼10–20% of diagnosed dementia cases overall.^4^ Pathologically, LBD is characterized by widespread cortical and subcortical deposition of misfolded α-synuclein aggregates that disrupt neuronal homeostasis and neurotransmission.^5,6^ Clinically, patients with LBD manifest a heterogeneous constellation of cognitive, neuropsychiatric, sleep-related and parkinsonian symptoms that complicate diagnosis and management.^7^

A defining, diagnostically valuable feature of LBD is cognitive fluctuations (CF), which are episodic, often rapid shifts in attention, alertness and cognitive performance. CF are considered a core diagnostic criterion and affect ∼60–80% of individuals with clinically defined LBD.^8^ CF significantly contribute to functional decline, caregiver burden and healthcare utilization.^8-11^ Converging evidence implicates disruptions in cholinergic and dopaminergic neurotransmission, and recent studies highlight dynamic network instability as a key mechanism underlying CF.^12^

Resting-state electroencephalography (EEG) studies consistently show that LBD is characterized by spectral slowing, including reduced posterior dominant frequency, increased theta/delta activity, reduced alpha activity and reactivity and increased dominant frequency variability. Quantitative EEG features have demonstrated value for distinguishing DLB from Alzheimer’s disease and for identifying prodromal Lewy body disease, and several studies report relationships between these spectral features and the clinical phenomenon of cognitive fluctuations.^13-15^ Contemporary mechanistic accounts further suggest that cognitive fluctuations reflect impaired stability and regulation of large-scale brain network states, motivating approaches that quantify not only ‘average’ spectral abnormalities but also the dynamics of state occupancy and switching.^12,16,17^

While previous studies demonstrated value of EEG in investigating LBD,^18,19^ EEG’s limited spatial resolution and poor sensitivity to deep cortical sources restrict its ability to localize neural abnormalities with precision.^15,20^ Functional MRI (fMRI), while providing whole-brain coverage, is constrained by low temporal resolution and reliance on delayed and indirect haemodynamic signals, limiting its ability to capture the rapid, sub-second neural transitions that underlie CF.^21^ Furthermore, conventional functional MRI approaches to dynamic functional connectivity (dFC) analysis, such as the sliding-window method, lack the temporal granularity required to resolve short-lived and recurrent brain states associated with CF.^22^

Magnetoencephalography (MEG) overcomes these limitations by providing millisecond-level temporal resolution combined with source-space reconstruction, offering non-invasive access to fast, large-scale cortical network dynamics.^23^ Hidden Markov model (HMM) analysis of MEG data enables detection of short-lived (∼100–200 ms), recurring ‘states’—distinct spatial and spectral configurations of large-scale network activity—without strong a priori assumptions.^24,25^ These capabilities make MEG with HMM particularly well-suited to interrogate the rapid, state-dependent neural dynamics hypothesized to underlie CF in LBD. To date, however, no studies have directly examined the neural mechanisms of CF using MEG, representing a significant gap in the literature.

The current study leverages resting-state MEG and HMM-based dFC analysis to uncover state-specific neural signatures of cognitive fluctuations in LBD. We addressed three key questions: (i) Do individuals with LBD exhibit a distinct pattern of transient brain states compared with patients with Parkinson’s disease (PD) without dementia and age-matched controls? (ii) Do specific brain states show frequency-specific spectral slowing—indexed by elevated theta/beta power ratio (TBR)—in anatomically localized cortical regions (extending prior EEG findings of global slowing into the source space^18,19^)? and (iii) Are these state- and region-specific spectral signatures correlated with the clinical severity of fluctuations, as measured by the Clinician Assessment of Fluctuation (CAF)? By integrating HMM-based dFC with high-temporal-resolution MEG, this work aims to reveal dynamic neural correlates of CF in LBD, which could inform the development of biomarkers for diagnosis, disease monitoring and therapy assessment in LBD.

## Materials and methods

### Ethics statement

This study was conducted in accordance with the Declaration of Helsinki and was approved by the Institutional Review Board (IRB) of the University of Florida. All participants at the University of Florida provided written informed consent under the approved IRB protocol. Additionally, MEG and MRI data from the Cambridge Centre for Ageing and Neuroscience (CamCAN) were used in accordance with ethical approval granted by the local research ethics committee at the University of Cambridge.

### Study participants

We prospectively recruited 31 participants at the Norman Fixel Institute for Neurological Diseases, University of Florida (UF) and assigned them to one of three groups:

- *LBD* (*n* = 7, age = 70 ± 6 years), including individuals clinically diagnosed with either DLB (*n* = 5) or PDD (*n* = 2). Diagnoses were made by fellowship-trained movement disorders specialists at the University of Florida based on established international criteria (i.e. the 2017 DLB consensus guidelines and the 2007 Movement Disorder Society criteria for PDD). Given the small size of the PDD subgroup, formal inferential comparisons between DLB and PDD, or inclusion of diagnostic subtype as a covariate, were not performed because such analyses would be statistically underpowered and potentially unreliable. Instead, descriptive subgroup summaries (DLB, PDD, PD and healthy controls) of fractional occupancy, state-resolved spectral power and theta-to-beta ratio are provided in Supplementary Figs 1 and 2.
- *Parkinson’s disease without dementia* (PD; *n* = 9, age = 73 ± 6 years), met Movement Disorder Society clinical diagnostic criteria for idiopathic Parkinson’s disease, without evidence of dementia.^26^ Cognitive assessments confirmed intact or only mildly impaired function.
- *Cognitively normal controls* (NC; *n* = 15, age = 72 ± 7 years), had no history of neurological or psychiatric conditions and scored within normal ranges on standard cognitive testing.

Medication exposure was recorded for all participants. All LBD participants (DLB and PDD) were treated with levodopa–carbidopa (Sinemet 25/100 mg) in combination with the cholinesterase inhibitor donepezil (10 mg), whereas PD participants without dementia were treated with levodopa–carbidopa only. No participants were receiving dopamine agonists, antipsychotics, sedatives/benzodiazepines, or antidepressants at the time of the study. Participants continued their usual medications, and MEG recordings were therefore acquired in a comparable practical ‘on-medication’ state. Given the modest sample size, additional medication covariate modelling or stratified sensitivity analyses were not performed; medication-related effects are therefore reported descriptively and considered in the interpretation of result.

To establish a broader normative reference for the HMM analysis, we included an additional 239 age-matched cognitively normal older adults (73 ± 7 years) from the publicly available Cambridge Centre for Ageing and Neuroscience (CamCAN) dataset.^27^ Demographic and clinical details are summarized in Table 1.

**Table 1:** Demographic and clinical data

Characteristic	LBD *n* = 7	NC *n* = 15	PD *n* = 9	*P*-value^a^
Age, years				0.74
Median (Q1–Q3)	70 (68–76)	73 (66–75)	76 (69–78)	
Education, years				0.82
Median (Q1–Q3)	16 (12–16)	16 (14–18)	16 (16–16)	
Disease duration, years				0.85
Median (Q1–Q3)	3 (1–3)	NA	2 (1–3)	
Sex, *n* (%)				0.22
F	2 (29)	11 (73)	5 (56)	
M	5 (71)	4(27)	4 (44)	
MoCA ^b^				< 0.001
Median (Q1–Q3)	17 (13–18)	25 (24–27)	23 (22–24)	
CDR-SB ^c^				< 0.001
Median (Q1–Q3)	6 (3–12)	0 (0–1)	1 (0–3)	
UPDRS Part III ^d^				0.24
Median (Q1–Q3)	33 (18–48)	NA	14 (13–22)	
CAF ^e^				0.008
Median (Q1–Q3)	6 (1–6)	NA	0 (0–0)	
RBD (MSQ Partner Q1) ^f^				0.74
*n* (%)	4 (57)	NA	3 (38)	
DoA (MSQ Partner Q8) ^g^				0.018
Median (Q1–Q3)	5 (4–7)	NA	9 (8–10)	
VH (NPI Q) ^h^				0.13
*n* (%)	3 (43)	NA	0 (0)	
CF (CAF > 5) ^i^				
*n* (%)	4(57)	NA	NA	

NA, not applicable.

^a^ *P*-values calculated using Kruskal–Wallis rank sum test for continuous variables and Fisher's exact test for categorical variables. All *P*-values are adjusted for false discovery rate (FDR). b MoCA: Montreal Cognitive Assessment. c CDR-SB: Clinical Dementia Rating—Sum of Boxes. d UPDRS Part III: Unified Parkinson’s Disease Rating Scale, Part III (Motor Examination). e CAF: Clinician Assessment of Fluctuation. f RBD: Rapid Eye Movement Sleep Behaviour Disorder (Mayo Sleep Questionnaire Partner Question 1). g DoA: Degree of Arousal (Mayo Sleep Questionnaire Partner Question 8). h VH: Visual Hallucinations (Neuropsychiatric Inventory Question). i Cognitive fluctuation: (CAF ≥ 5).

### Clinical measures

All prospectively recruited participants at UF completed the Montreal Cognitive Assessment (MoCA)^28^ and the Quick Dementia Rating System.^29^ The Quick Dementia Rating System is an informant-reported questionnaire from which a Clinical Dementia Rating (CDR) Dementia Staging Instrument sum of boxes score (CDR-SB) can be reliably estimated. Individuals with LBD and PD also underwent the motor examination of the Unified Parkinson Disease Rating Scale. For LBD/PD cohorts, study partners completed CAF,^30^ One Day Fluctuation Scale,^30^ the Mayo Fluctuations Scale,^31^ Neuropsychiatric Inventory Questionnaire,^32,33^ and the National Alzheimer Coordinating Center version of the Mayo Sleep Questionnaire.^34^ Demographics and medical history were also collected. The CAF was chosen as the primary measure of fluctuations for the current study because it reflects a 1-month period (as opposed to the One Day Fluctuation Scale, which could miss fluctuations not occurring on the day prior to assessment) and includes a continuous 0–12 severity scale (as opposed to the Mayo Fluctuations Scale which is a 4-point screening measure).

### MEG data acquisition and preprocessing

MEG recordings were acquired using a 306-channel Elekta/MEGIN TRIUX system (204 planar gradiometers and 102 magnetometers) housed in magnetically shielded rooms at both UF and the University of Cambridge. The CamCAN data were acquired in a single ∼10-min session during eyes-closed resting-state condition. At UF, resting-state data were collected in five alternating eyes-open and eyes-closed sessions, each lasting over 2 min. For consistency with CamCAN, only the eyes-closed sessions were included in our analysis. To minimize vigilance-related confounding during eyes-closed resting-state MEG, UF recordings were acquired in short (∼2 min) alternating eyes-open/eyes-closed blocks. If a participant appeared drowsy, acquisition was paused and resumed after brief interaction to restore alertness. For the CamCAN dataset, which includes a longer continuous eyes-closed resting run, analyses were restricted to the first 120 s to reduce the likelihood of progressive drowsiness during prolonged eyes-closed recording.

Raw MEG data were preprocessed using temporal signal space separation(tSSS)^35,36^ via the MaxFilter software (MEGIN Oy, Helsinki, Finland) to suppress external noise and artefacts. MEG recordings were processed using the Oxford Centre for Human Brain Activity (OHBA) Software Library for the Analysis of Electrophysiology Data (osl-ephys),^25,37^ which builds on MNE-Python.^38^ MEG data were then band-pass filtered (0.5–125 Hz) and notch filtered to remove powerline noise. Bad channels were identified using variance-based outlier detection and interpolated. Independent component analysis was performed to remove artefacts, using cross-trial phase statistics with a Kuiper threshold of 0.8.^39^ Bad segments were detected and excluded using amplitude- and variance-based criteria. Finally, MEG data were downsampled to 250 Hz.

From each session, the first 120 s of artefact-free data were retained for further analysis. After removing sessions with excessive artefacts and/or noise, a total of 151 sessions (LBD: 34, PD: 44, NC: 73) from the UF data and 239 CamCAN sessions were included, yielding 390 sessions for analysis.

Individual T_1_-weighted structural MRI data were available for all participants from both cohorts (CamCAN and UF) and were used for subject-specific MEG source modelling. All T_1_-weighted MRIs were processed using the same standardized pipeline to ensure consistency across cohorts. T_1_-weighted MRIs were processed using FSL’s Brain Extraction Tool and FMRIB's Automated Segmentation Tool to derive scalp and brain surfaces.^40,41^ MEG-MRI co-registration was performed using osl-ephys’s RHINO (Registration of head shapes including nose) pipeline. A rigid-body surface-matching algorithm was then applied to align MEG and MRI space. Single-shell boundary-element models were constructed from each subject’s scalp surface to compute the lead field matrix.^42^

Linearly constrained minimum variance beamforming was used to reconstruct source activity in the 1–80 Hz band, combining data from magnetometers and gradiometers. The sensor covariance matrix was regularized by fixing the data rank at 60.^43^ Source-space time series were projected onto the Glasser 52 cortical parcels (MNI152NLin6, 8 mm resolution) using a spatial-basis approach.^44^ To mitigate signal leakage, symmetric orthogonalization was applied across parcels.^45^ The resulting parcel-wise time series provided a low-dimensional representation of whole-brain MEG dynamics for subsequent connectivity and network analyses.

### Hidden Markov models

We used hidden Markov models (HMMs) to identify short-lived, recurring patterns of large-scale cortical activity (‘brain states’) directly from MEG time series. In MEG-HMM analyses, a brain state is a transient configuration in which specific cortical parcels co-activate and co-oscillate with a characteristic spectral/covariance ‘fingerprint’; the brain rapidly transitions among such configurations on sub-second timescales (typically ∼100–200 ms during rest). HMMs recover these states without pre-defining time windows or networks, allowing us to study when particular configurations appear and how long they persist. For interpretability, we summarize the state time course using standard metrics (e.g. fractional occupancy and mean lifetime) with formal definitions and statistical treatment provided in Data Analysis Section.^24,46^

Formally, the HMM specifies the joint distribution over observations $x_{1:T}=(x_{1},\ldots ,x_{T})$ and latent states $\theta_{1:T}=(\theta_{1},\theta_{2},\ldots ,\theta_{T})$ as


(1) $$p(x_{1:T},\theta_{1:T})=p(\theta_{1})\;p(x_{1}|\theta_{1})\overset{T}{\underset{t=2}{\prod }}\;p(\theta_{t}|\theta_{t-1})\;p(x_{t}|\theta_{t})$$

Here, each state $\theta_{t}\in \{1,\ldots ,K\}$ evolves via a transition matrix *A*, where


(2) $$p(\theta_{t}=j\;|\;\theta_{t-1}=i)=A_{ij}\;$$

and each observation $x_{t}\in R^{D}$ is drawn from a state-conditional emission distribution, which are typically modelled as multivariate Gaussians,


(3) $$p(x_{t}\;|\;\theta_{t}=k)=N(x_{t}\;|\;\mu_{k},\Sigma_{k})$$

with state-specific mean $\mu_{k}$ and covariance $\Sigma_{k}$. This formulation allows HMMs to capture both changes in network activation (via $\mu_{k}$) and functional connectivity patterns (via $\Sigma_{k}$).^25,47^

Exact inference in HMMs, which is involves computing the posterior over $\theta_{1:T}$ and model parameters, is intractable for high-dimensional data; thus, practical methods rely on approximations. The classical approach is the Baum-Welch algorithm, an instance of expectation-maximization that uses forward-backward recursions to compute sufficient statistics for maximum-likelihood estimation of *A*, $\mu_{k},\Sigma_{k}$ and the most likely state sequence.^48,49^ To quantify uncertainty in parameters, variational Bayes (VB) introduces a factorized surrogate posterior (*q*) and maximizes a lower bound on the model evidence, trading exactness for tractability.^50^ For large neuroimaging datasets, stochastic variational inference scales variational Bayes by performing noisy natural-gradient updates on mini-batches of data, enabling efficient convergence without loading the entire dataset into memory.^51^

#### Time-delay embedded HMM

To make the HMM sensitive to oscillatory and transient coupling phenomena present in the raw electrophysiological signal (beyond slower power-envelope co-modulations), we used a time-delay embedded HMM (TDE-HMM). Time-delay embedding augments each time point with multiple lagged copies of the signal to reconstruct the local state-space dynamics; fitting a Gaussian-emission HMM to this embedded space allows states to be characterized by spectrally resolved covariance structure (power and phase-locking) and to capture rapid transitions. This approach has been widely used in MEG to recover fast, recurring, frequency-specific states.^46,52,53^

#### Implementation

We used the OSL-Dynamics toolbox (Oxford Centre for Human Brain Activity, University of Oxford) for TDE-HMM fitting.^37^ Parcel-level source time series from all participants were temporally concatenated. Signals were standardized; then a time-delay embedding with 15 lags (∼60 ms) was applied (original sample plus 15 immediate past values), enhancing sensitivity to low-frequency content and transient bursts. To control dimensionality, we applied principal component analysis to the embedded data and retained 120 principal components. Processed sequences were saved as TFRecord files with a fixed sequence length of 400 samples for efficient mini-batch training.

The HMM was configured with *K* = 6 states, consistent with prior resting-state MEG work reporting 4–8 states.^24^ In MEG/EEG HMM analyses, the number of states (*K*) must be specified a priori. Although the variational free-energy objective provides an approximation to model evidence, prior MEG-HMM work shows that free energy often continues to improve with increasing *K*, so a single ‘optimal’ peak is frequently absent^52^; consequently, *K* is typically chosen to balance interpretability against redundancy and estimation reliability.^24,52,54^ We therefore adopted *K* = 6 as a parsimonious and commonly used resolution for resting-state MEG TDE-HMM analyses, yielding well-separated, non-redundant states with sufficient occupancy for stable spectral estimation.^24,52^

During HMM training, state covariances and transition probabilities were learned while state means were fixed (standard in TDE-HMM to emphasize covariance structure). We optimized a variational free-energy objective using mini-batches (batch size = 32, learning rate = 0.001) for 10 epochs. To mitigate suboptimal local minima, we performed a short random-initialization phase (three 1-epoch runs), then repeated training five times with different seeds and selected the solution with the lowest variational free energy for downstream analyses. See Data Analysis Section for how we computed fractional occupancy, mean lifetime and transition statistics from the posterior state time courses.

### Data analysis

After fitting an TDE-HMM, we quantified the temporal characteristics of each hidden state using summary metrics, including fractional occupancy (proportion of time spent in each state), mean lifetime (average duration of a state visit) and mean interval (average duration between successive visits to the same state).^24,25^ These metrics were computed at the individual subject level and across groups. Due to the high interdependence among FO, mean lifetime and mean interval, we focused on FO as the primary indicator of temporal engagement with each state in this study.

To characterize oscillatory activity, we estimated power spectral density for each state using the multitaper approach.^55^ Timepoints were weighted by the posterior state probability, segmented into non-overlapping windows (2× sampling rate for 0.5 Hz resolution), tapered using orthogonal Slepian functions and Fourier transformed. Power spectral densities were averaged across tapers and windows to yield state-specific spectra.^52^ Theta/beta power ratios (TBRs) were calculated per region and state to assess slowing. Frequency bands were defined as: *δ* band (<4 Hz), *θ* band (4 Hz ≤ frequency < 8 Hz), *α* band (8 Hz ≤ Frequency < 13 Hz) and *β* band (13 Hz ≤ Frequency < 30 Hz).

Group-level differences in fractional occupancy across the six states were assessed using nonparametric permutation *t*-tests. For each comparison, 5000 label permutations were performed to generate empirical null distributions, enabling inference without distributional assumptions. Multiple state-wise comparisons were controlled using Bonferroni correction.

State-dependent spectral slowing was quantified using TBR computed for all region-of-interest (ROI) × state combinations. Group differences in spectral power and TBR among LBD, PD and NC groups were evaluated using permutation-based general linear models (5000 label shuffles), with age, sex and years of education included as covariates. Multiple-comparison correction was applied across the State × ROI × Frequency Band space using the Benjamini–Hochberg false discovery rate (FDR) procedure. Effect sizes were quantified using Hedges’ *g*.

To assess whether state-specific slowing effects were independent of global power shifts, we conducted a sensitivity analysis in which subject-wise global band power was included as an additional covariate. For each frequency band, global power was defined as the mean spectral power across all cortical parcels. Group effects were evaluated using covariate-adjusted models, with statistical significance determined via permutation testing and appropriate correction for multiple comparisons. This residualization approach ensured that regional and state-specific effects were not driven by whole-head power differences.

Within the LBD group, associations between spectral abnormalities and cognitive fluctuations were examined using permutation-based Spearman correlations (5000 permutations) between CAF scores and state-resolved spectral power across ROIs and frequency bands. FDR correction was applied within each state–frequency band. Because Spearman’s *ρ* directly reflects effect size, it is reported as both the measure of association and effect size. The robustness of significant correlations was further evaluated using jackknife leave-one-out analyses; ROIs were considered robust if they met all of the following criteria: FDR-corrected *P* < 0.05, 100% sign consistency across jackknife iterations and absolute Spearman’s *ρ* exceeding the jackknife standard error.

All statistical computations were performed using R (v4.4.2). Visualization of the statistical results was performed via ggseg,^56^ employing custom functions (e.g. *geom_brain*) to project the findings onto brain surfaces defined by the *Glasser52* atlas.

## Results

### Clinical and demographic characteristics

Table 1 presents clinical and demographic characteristics of LBD, PD and NC groups. Groups did not differ in age, sex and educational attainment (*P* > 0.05). As expected, participants with LBD scored significantly (*P* < 0.05) higher in CAF (median: 6) than PD (median: 0), consistent with the scale’s discriminative validity for fluctuating cognition. Cognitive performance followed the anticipated gradient LBD < PD < NC (MoCA mean ± STD: 16 ± 6 versus 23 ± 1 versus 25 ± 2; *P* < 0.001). Functional impairment mirrored this pattern (CDR-SB 7.3 ± 4.6 versus 1.3 ± 1.5 versus 0.3 ± 0.4; *P* < 0.001).

### Group differences in brain state occupancy

**Figure 1:**
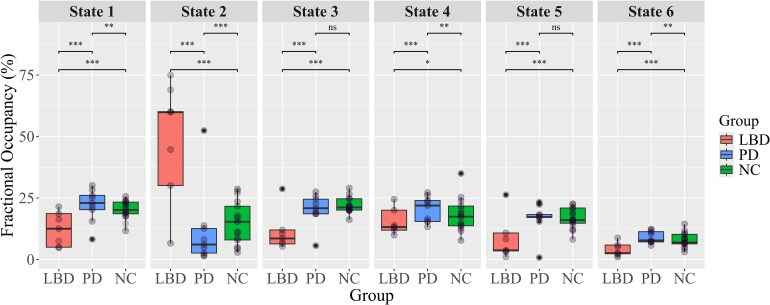
Altered brain state dynamics in Lewy body dementia identified by hidden Markov modelling. Boxplots show the fractional occupancy (FO) of the six brain states derived from time-delay embedded hidden Markov modelling (TDE-HMM) across three groups: Lewy body dementia (LBD, *n* = 7 participants), Parkinson’s disease without dementia (PD, *n* = 9 participants) and cognitively normal controls (NC, *n* = 15 participants from the prospective UF cohort; an additional 239 NC from the CamCAN dataset were used for HMM training but not for group statistical comparisons). The experimental unit is the individual participant, and each datapoint represents one independent subject’s mean fractional occupancy across the five sessions for a given state. Group differences in fractional occupancy for each state were assessed using nonparametric permutation t-tests (5000 label permutations). Significant differences are indicated with asterisks (*P < 0.05, **P < 0.01, ***P < 0.001, Bonferroni-corrected for multiple states; ‘n.s.’: not significant). For LBD versus NC/PD in State 2, permutation t-tests yielded *P* < 0.001. LBD participants exhibited significantly increased FO in State 2 and reduced FO in State 6 compared with both NC and PD groups. PD participants showed the highest FO in State 1, while NC participants demonstrated a more balanced distribution with higher occupancy in States 3 and 5.

Figure 1 shows fractional occupancy (FO) of six brain states across the three groups. Distinct group-specific patterns emerged, particularly in States 2 and 6. Participants with LBD showed significantly increased FO in State 2 compared to both NC and PD groups (*P* < 0.001 for both), suggesting LBD-specific neural engagement. In contrast, State 6 had the lowest FO in LBD, significantly smaller than both NC and PD (*P* < 0.001).

Participants with PD showed the highest FO in State 1, exceeding NC (*P* < 0.001) and LBD (*P* < 0.001), with NC also higher than LBD (*P* < 0.001), indicating PD-related state preference. In States 3 and 5, NC participants showed elevated FO compared to both LBD and PD (*P* < 0.001); PD also exceeded LBD in State 5 occupancy (*P* < 0.01). Differences in State 4 were more modest, reaching significance only between NC and LBD (*P* < 0.05).

Collectively, LBD was characterized by dominant engagement with State 2 and minimal occupancy of State 6, while NC displayed dominant occupancy in States 3 and 5. These patterns highlight distinct neural state profiles across groups, with NC and PD exhibiting more balanced FO distributions than LBD.

### Spectral slowing and theta/beta power ratios

**Figure 2:**
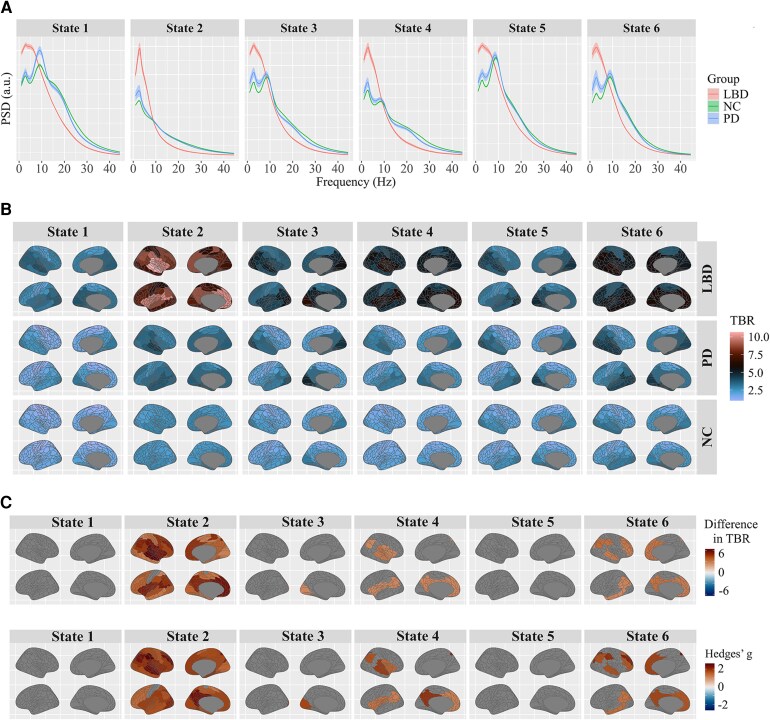
Spectral slowing in LBD and regional theta/beta power ratios (TBRs) across brain states. ((**A**) Power spectral density (PSD) plots averaged across participants for the six brain states in Lewy body dementia (*n* = 7), Parkinson’s disease without dementia (*n* = 9) and cognitively normal controls (*n* = 15) groups. The experimental unit is the individual participant. (**B**) Cortical surface maps (Glasser 52 atlas) display mean TBR distributions across the six brain states in each group. States 2 and 6 showed the most prominent slowing in LBD. (**C**) Group-level differences in TBR (LBD versus NC) across the six brain states after adjustment for age, sex and years of education, with corresponding effect sizes (Hedges’ g). TBR differences were evaluated using permutation-based general linear models (5000 label shuffles) with covariates (age, sex, education). Multiple comparisons were controlled usingfFalse discovery rate (FDR) across the state × ROI × band space. States 2 and 6 showed the largest TBR increases in LBD relative to NC (e.g. State 2 overall TBR: LBD mean ± SD 7.79 ± 3.66 versus NC 2.46 ± 0.66, permutation-based *P* < 0.001; similar for State 6, *P* < 0.001). Effect sizes were medium-to-large.

Figure 2A shows the power spectral density across the six brain states for participants in the LBD, PD and NC groups. Participants with LBD exhibited pronounced spectral slowing, reflected by significantly elevated TBRs, particularly in States 2 and 6. In State 2, the TBR in the LBD group (mean ± SD: 7.79 ± 3.66) was significantly higher than that in the NC (2.46 ± 0.66) and PD (3.19 ± 2.29) groups (*P* < 0.001, for both comparisons). A similar pattern was observed in State 6, where participants with LBD exhibited significantly elevated TBRs (4.91 ± 1.44) relative to NC (2.21 ± 0.85) and PD (2.96 ± 1.94) participants (*P* < 0.001, for both comparisons). While additional group differences were noted in other states, TBR differences between NC and PD were generally non-significant, with the exception of State 5 (*P* < 0.05). These findings suggest that elevated low-frequency activity, as reflected by TBR, is a specific neurophysiological hallmark of LBD rather than a generalized feature of Parkinsonian syndromes.

Figure 2B highlights cortical regions showing the most prominent spectral slowing in LBD during States 2 and 6. Qualitatively, State 2 was characterized by greater involvement of frontoparietal and anterior cingulate/medial prefrontal regions, as well as auditory association cortex, whereas State 6 emphasized posterior cingulate/precuneus and visual–parietal regions. These spatial patterns provide functional context for the two states most strongly associated with LBD-related group differences.

The left anterior cingulate and medial prefrontal cortex (ACC and mPFC) consistently showed the highest TBR (State 2: 10.0 ± 6.3; State 6: 6.1 ± 3.0), followed by the left posterior cingulate (State 2: 9.7 ± 5.7; State 6: 5.3 ± 2.2) and right dorsolateral prefrontal cortex (State 2: 8.6 ± 4.1; State 6: 5.4 ± 2.4). The right dorsal stream visual cortex also showed prominent slowing.

Several ROIs exhibited greater slowing specifically in State 2, including the right early auditory cortex (10.6 ± 5.3), right auditory association cortex (10.7 ± 4.8) and left temporal-parieto-occipital junction (7.2 ± 3.3). In contrast, State 6 prominently involved visual and attentional processing regions, such as the right ventral stream visual cortex (5.8 ± 3.5), left medial temporal cortex (5.8 ± 3.0), right inferior parietal cortex (IPC-task-positive; 5.3 ± 2.2) and right insular-frontoparietal operculum cortex (5.2 ± 1.9). These findings highlight a robust and state-dependent pattern of spectral slowing in LBD, supporting its role as a distinct neural signature of the disease.

**Figure 3:**
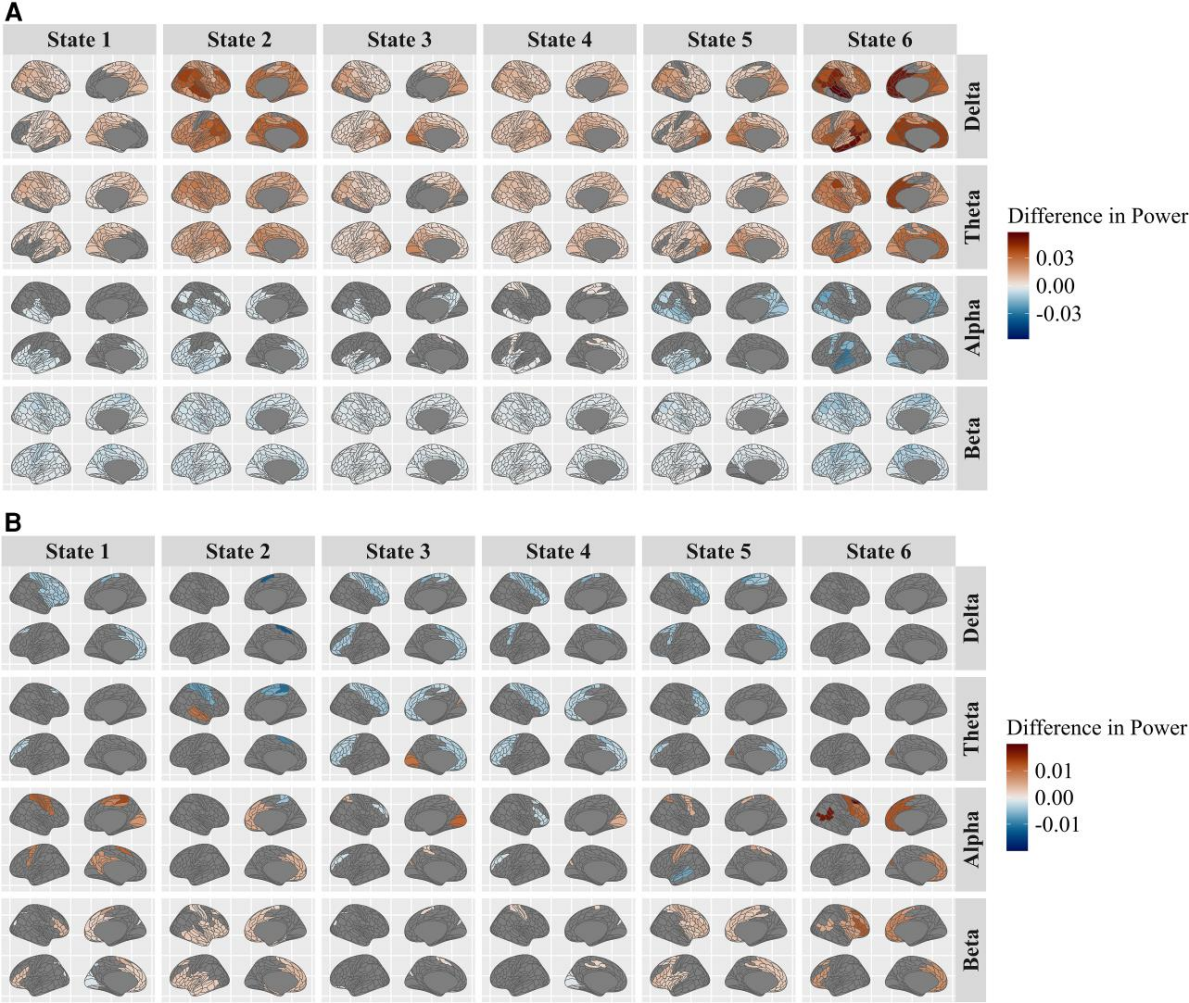
Group differences in the spatial distribution of regional spectral power (adjusted for age, sex and years of education). Cortical surface maps illustrate statistically significant differences in regional spectral power (delta/theta increases in warm colours; alpha/beta decreases in cool colours) between groups across the six HMM-derived brain states. (**A**) LBD (*n* = 7) versus NC (*n* = 15). (**B**) PD (*n* = 9) versus NC (*n* = 15). The experimental unit is the individual participant. Analyses used permutation-based general linear models (5000 label shuffles) with FDR correction across the state × ROI × frequency band space. Significant clusters (FDR-corrected *P* < 0.05) are displayed. In LBD versus NC, States 2 and 6 exhibited the most marked low-frequency power increases (e.g. in ACC/mPFC, visual cortices) and high-frequency reductions.

After adjustment for age, sex and education, group differences in TBR between LBD and cognitively NC remained robust (Fig. 3C). Sensitivity analyses accounting for subject-wise global power, as well as additional group comparisons, are presented in Supplementary Figs 3–6. Effect sizes (Hedges’ *g*) are reported for all contrasts and consistently indicate medium-to-large effects, in line with the primary analyses. Importantly, inclusion of global power as a covariate did not alter the overall pattern of results: States 2 and 6 continued to exhibit the most pronounced group differences. Together, these findings indicate that the observed spectral slowing is not driven by a nonspecific global power shift, but instead reflects state-specific neurophysiological alterations associated with LBD.

### Regional spectral power in LBD

Spectral power analyses revealed consistent differences between LBD and NC participants across frequency bands and brain states. LBD showed increased power in lower-frequency bands (δ,θ) and reduced power in higher-frequency bands (α,β), consistent with neurodegenerative slowing (Fig. 3A).

In State 2, LBD exhibited elevated *δ* and *θ* power in multiple regions, including the ACC/mPFC (*δ*: 0.016; *P* < 0.01), right medial intraparietal sulcus (*δ*: 0.020; *P* < 0.01), left dorsal stream visual cortex (*δ*: 0.020; *P* < 0.01) and right inferior dorsolateral prefrontal cortex (*δ*: 0.019; *P* < 0.01). Additionally, the left IPC, right dorsal stream visual cortex, left early auditory cortex, primary and early visual cortex, lateral temporal cortex and superior medial parietal cortex showed increased power in *δ* and *θ* bands, reflecting widespread involvement of sensory, associative and executive regions.

In State 6, LBD showed reduced *α* and *β* power, most prominently in the right IPC (*α*: −0.019; *P* < 0.01) and left intraparietal sulcus and PGP (*α*: −0.014; *P* < 0.05). Additionally, reduction in the power of *α* and *β* power bands observed bilaterally in dorsal stream visual, auditory and primary visual cortices, as well as the posterior cingulate.

Compared with NC, participants with PD showed smaller but significant differences, primarily in State 6 (Fig. 3B). Power of the *δ* band increased in several regions in the left hemisphere, including the superior dorsolateral prefrontal cortex, lateral temporal cortex, ventral stream visual cortex, intraparietal sulcus and posterior parietal gyri (IPS/PGP), anterior cingulate and medial temporal cortex. Conversely, *α* and *β* power was reduced in PD across regions such as the right IPS/PGP, left middle temporal + complex, suggested to contain both middle temporal and medial superior temporal,^57^ right dorsal stream visual cortex, left IPC (task-negative and task-positive networks), auditory association cortex, temporal-parieto-occipital junction and bilateral medial intraparietal sulci (*P* < 0.05).

### Association between spectral power and cognitive fluctuations

**Figure 4:**
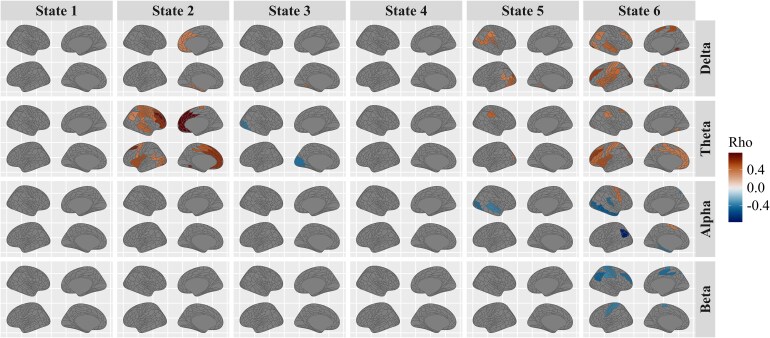
Robust neural correlates of cognitive fluctuations in LBD identified by jackknife analysis. Cortical surface maps show Spearman correlations (*ρ*) between Clinician Assessment of Fluctuation (CAF) scores and regional spectral power across frequency bands and brain states within the LBD group (*n* = 7 participants; experimental unit = individual participant). Only robust ROIs surviving jackknife leave-one-out resampling are displayed (FDR-corrected *P* < 0.05 within each state–frequency band, 100% sign consistency across iterations and |*ρ*| > jackknife standard error). Prominent examples include positive θ-band correlation in left ventral stream visual cortex during State 2 (*ρ* = 0.65, *P* < 0.001) and negative α-band correlations in superior medial parietal cortex during State 6 (left: *ρ* = −0.64, *P* < 0.001; right: *ρ* = −0.56, *P* < 0.001). Jackknife-derived standard errors and additional correlations with MFS/ODF scales are in Supplementary Figs 7–9.

Figure 4 shows the associations between CAF scores and regional spectral power across frequency bands and brain states. The jackknife-derived standard errors for the robust CAF correlations, along with the significant Mayo Fluctuations Scale and One Day Fluctuation Scale correlations, are reported in Supplementary Figs 7–9. Overall, CAF scores exhibited a consistent pattern of positive correlations with lower frequency bands (*δ* and *θ*) and negative correlations with higher frequency bands (*α* and *β*), with these associations being most prominent in brain States 2 and 6.

In State 2, significant positive correlations in the *θ* band were observed in multiple cortical regions. These included the left ventral stream visual cortex (*ρ* = 0.65; *P* < 0.001), left supplementary motor area (*ρ* = 0.48; *P* < 0.05) and bilateral superior dorsolateral prefrontal cortex (*ρ* = 0.54; *P* < 0.05). Additional correlations were found in the left temporal-parieto-occipital junction (*ρ* = 0.36; *P* < 0.05) and the right inferior frontal cortex (*ρ* = 0.60; *P* < 0.05). The right auditory association cortex also showed a moderate positive correlation in the *θ* band (*ρ* = 0.43; *P* < 0.05).

In State 6, positive correlations were identified between CAF scores and *δ* or *θ* power in in the left ventral stream visual cortex (*δ*: *ρ* = 0.52; *P* < 0.05) and the task-positive IPC, which showed positive correlation in the left hemisphere (*θ*: *ρ* = 0.47; *P* < 0.05) and negative correlation in the right hemisphere (*β*: *ρ* = −0.37; *P* < 0.005). The task-negative IPC showed strong negative correlations in the *α* band (left: *ρ* = −0.72; *P* < 0.001) and *β* band (right: *ρ* = −0.48; *P* < 0.001).

Other key regions exhibiting strong correlations with cognitive fluctuations in State 6 included the left medial bank of the intraparietal sulcus showed strong positive correlations in both *δ* (*ρ* = 0.42; *P* < 0.05) and *θ* bands (*ρ* = 0.50; *P* < 0.05). In the superior somatosensory and motor cortex, the left hemisphere demonstrated a positive *δ* correlation (*ρ* = 0.35; *P* < 0.05) and a negative *β* correlation (*ρ* = −0.37; *P* < 0.05), while the right hemisphere showed a *β* band negative correlation (*ρ* = −0.38; *P* < 0.05). The superior medial parietal cortex exhibited *α*-band negative correlations (left: *ρ* = −0.64; *P* < 0.001; right: *ρ* = −0.56; *P* < 0.001). In addition, the left cingulate motor area and area 5 exhibited a positive correlation in *θ* band (*ρ* = 0.37; *P* < 0.05).

In state 5, the left ventral stream visual cortex showed negative correlations in *α* band (*ρ* = −0.37; *P* < 0.05). The middle temporal + complex and adjacent visual areas showed a strong positive correlation with CAF in the *δ* band (*ρ* = 0.45; *P* < 0.05) in the left hemisphere, whereas the right hemisphere demonstrated a negative correlation in the *α* band (*ρ* = −0.43; *P* < 0.05).

These results highlight a complex, state- and region-specific pattern of associations between CAF scores and neural oscillatory dynamics in LBD. They suggest that cognitive fluctuations are linked to both heightened low-frequency activity and reduced high-frequency power in distributed cortical networks.

## Discussion

Leveraging the high spatiotemporal resolution of MEG and advanced HMM, our study reveals distinct dynamic, state-dependent neural signatures underlying cognitive fluctuations in LBD. We identified distinct electrophysiological features of LBD (e.g. aberrant state occupancy, widespread spectral slowing and direct correlations with clinical fluctuation scores) that reliably distinguish LBD from both PD without dementia and cognitively intact controls. Taken together, these findings offer novel mechanistic insights into the neural basis of LBD and one of its core clinical features, cognitive fluctuations.

### State-specific neural signatures of LBD

A key finding of the current study is the imbalance in brain state dynamics in LBD. Specifically, patients with LBD exhibited significantly elevated fractional occupancy of State 2 and minimal engagement with State 6, in contrast to the more balanced state distributions observed in PD and cognitively NC. This aberrant pattern highlights disrupted network stability and may underlie the hallmark symptom of cognitive fluctuations in LBD.

From a neurobiological perspective, the spatial distribution of state-resolved spectral effects provides functional context for these findings. State 2 preferentially engaged an attention/control–related configuration, encompassing ACC/mPFC–frontoparietal and auditory association regions, whereas State 6 was dominated by posterior midline and visual–parietal regions, including the PCC/precuneus. These systems have been consistently implicated in attentional stability, arousal regulation and large-scale network switching in Lewy body dementias. Prior work further demonstrates that abnormalities in fast-timescale brain dynamics (captured by microstate- and state-based measures) are closely linked to the severity of cognitive fluctuations.^12,17,58,59^

These findings align with previous studies showing that dynamic connectivity analysis can reveal transient shifts in network activation patterns that are undetectable using static functional connectivity approaches^37,60,61^ Static connectivity assumes temporal stationarity and thus fails to capture the brain's intrinsic variability. In contrast, dFC, as implemented through our hidden Markov model, allows for the identification of rapid and discrete connectivity states with high temporal precision.

Traditional dFC methods, such as sliding-window techniques, require a fixed temporal resolution, creating a trade-off between temporal sensitivity and signal reliability.^62^ Moreover, such methods are limited in their ability to resolve brief, yet meaningful, fluctuations in connectivity, especially in short resting-state scans.^63^ Our HMM-based approach overcomes these limitations by inferring latent connectivity states directly from the full time series without the need for predefined windows.^64^ This yields temporally precise and neurobiologically interpretable brain states that better capture the dynamic nature of LBD-related network dysfunction.^65^

Although individual HMM state visits occur on sub-second timescales (∼100–200 ms), the clinically relevant metrics derived from these events (such as fractional occupancy, mean dwell time and switching rates) summarize the aggregate statistics of thousands of state visits over the full recording. As such, these measures index an individual’s propensity to enter and remain in particular network configurations over minutes of observation, providing a plausible link to slower, clinically observable fluctuations in attention and arousal. This multiscale view is supported by electrophysiological work using EEG microstates, which demonstrates long-range/scale-free temporal structure in sequences of brief states and shows that slowing of microstate dynamics in LBD relates to fluctuation severity.^66-68^ Within this framework, persistent shifts in moment-to-moment state expression and their associated spectral profiles may accumulate over time and manifest clinically as fluctuations in cognition and alertness on the order of minutes to hours.

### Spectral slowing as a biomarker of LBD pathophysiology

Our results demonstrate that spectral slowing, measured by elevated TBRs, is a robust and widespread electrophysiological feature of LBD that distinguishes it from both PD without dementia and cognitively healthy controls. Although spectral slowing was observed across all brain states, it was most pronounced in States 2 and 6, suggesting that specific dynamic network configurations may be more vulnerable to the underlying pathophysiological processes of LBD.

We observed a pronounced spectral slowing in the ACC/mPFC, regions that are critical for attentional control, inhibitory regulation and executive functioning. These regions consistently exhibited high TBR values, particularly in brain states linked to cognitive fluctuation severity. Our results align with prior reports showing increased TBR in LBD relative to both healthy controls and patients with AD, supporting spectral slowing as a translatable electrophysiological biomarker of LBD pathophysiology.^18^ Similarly, Schumacher *et al*. identified elevated pre-alpha (5.5–8 Hz) power and reduced beta activity as defining features in MCI due to LBD compared to MCI due to AD, reinforcing the diagnostic potential of frequency-specific slowing.^69^

Importantly, our study demonstrates that these spectral abnormalities are not static, but rather state-dependent, emerging prominently during transient brain states associated with altered functional configurations. This dynamic profile supports the hypothesis that cognitive fluctuations in LBD arise from unstable network dynamics rather than persistent circuit dysfunction, echoing findings by Stylianou *et al*.,^13^ who identified transient EEG markers as correlates of cognitive fluctuations in DLB.

Furthermore, visual network regions exhibited widespread oscillatory abnormalities closely associated with cognitive fluctuations. Elevated theta power in the primary and early visual cortices was significantly correlated with CAF scores (*ρ* = 0.36). The ventral stream visual cortex, crucial for object recognition,^70^ showed high TBR and significant positive correlations with CAF scores. These findings are consistent with prior studies reporting reduced occipital activation and perfusion deficits in LBD.^71,72^

Beyond visual and default-mode areas, we identified substantial oscillatory disruptions in frontal executive, auditory, sensorimotor, parietal attention and temporal-parietal junction regions. In State 2, the right auditory association cortex and early auditory cortex exhibited high TBR values (10.7 ± 4.8 and 10.6 ± 5.3, respectively), while the left supplementary motor area showed strong positive correlations with CAF scores in the theta band (*ρ* = 0.48). These findings underscore the diffuse yet regionally specific nature of cortical slowing in LBD, which may reflect a global vulnerability of high-order cognitive networks in this condition.

We acknowledge that global cognitive severity, as indexed by the MoCA, may contribute to overall spectral slowing in LBD. However, dementia severity is a defining clinical characteristic of LBD and is intrinsically linked to disease status. As such, adjusting for MoCA in primary case–control contrasts would amount to conditioning on an intermediate variable along the disease pathway, which may introduce overadjustment bias when estimating the total effect of diagnostic group on electrophysiological measures. In addition, the limited overlap in MoCA scores between demented and non-demented groups can result in unstable and difficult-to-interpret model estimates. Importantly, our results extend beyond time-averaged spectral differences and emphasize state-dynamic features—such as fractional occupancy and state-accentuated spectral slowing—that capture temporally structured alterations in brain network dynamics and provide information complementary to global cognitive severity, rather than reflecting generalized impairment alone.

### Neural correlates of clinical fluctuations

We found a correlation between regional spectral power and CAF scores, providing a direct neurophysiological correlation of cognitive fluctuations in LBD. We consistently observed positive correlations with low-frequency power (delta and theta) and negative correlations with high frequency power (alpha and beta) across key cortical regions. This pattern supports the hypothesis that cognitive fluctuations in LBD stem from intermittent disruptions in cortical arousal and network engagement.

Clinically, participants with LBD demonstrated significantly elevated CAF scores relative to PD, reflecting the disorder’s hallmark fluctuating cognition.^12,16^ As expected, MoCA scores were lowest in LBD versus PD and NC. While individual item performance was not assessed in the current study, attentional and executive deficits are particularly common in LBD.^73,74^ Finally, CDR-SB scores in LBD exceeded those in PD, reflecting greater global functional decline, as noted in other cohorts.^75^

The direct association between MEG-derived oscillatory dynamics and CAF severity suggests that quantitative MEG spectral measures could serve as objective markers of this core clinical feature of LBD. Notably, studies using resting-state EEG in autopsy-confirmed cohorts (e.g. Choi *et al*.^76^) have demonstrated that global theta power increases predict cognitive decline, reinforcing the clinical relevance of slow-wave oscillations as cognitive biomarkers.

### Clinical implications

The findings of this study have several potential clinical implications, particularly for the diagnosis, monitoring and management of LBD. LBD is associated with a distinct neurophysiological profile, characterized by increased occupancy of State 2, reduced engagement with State 6 and widespread spectral slowing. This profile may serve as a promising objective biomarker to help differentiate LBD from other neurodegenerative disorders.

Our results support the growing utility of MEG-based measures as non-invasive, high-resolution biomarkers of LBD. Elevated TBR in specific regions, particularly the ACC/mPFC, may serve as electrophysiological signature of LBD. These findings are align with previous works by van der Zande *et al*.^14^ and Dauwan *et al*.,^77^ who demonstrated the diagnostic value of EEG in prodromal DLB and in differentiating DLB from AD. MEG, with its superior spatial and temporal resolution, offers an even more sensitive platform for such assessments.

Crucially, the strong correlation between CAF scores and regional spectral power further highlights the potential of MEG-derived spectral metrics as objective and quantifiable markers of cognitive fluctuation severity. Traditional clinical assessments are limited in their ability to capture the transient and unpredictable nature of cognitive fluctuations, which are often episodic and underreported. Our results indicate that power ratios, particularly those reflecting shifts between low-frequency (*δ*, *θ*) and high-frequency (*α*, *β*) bands, may provide a temporally sensitive and biologically grounded index of fluctuating cognition.

### Limitations and future directions

While this study offers novel insights into the dynamic neurophysiological mechanisms underlying cognitive fluctuations in LBD, several limitations warrant consideration and guide directions for future research.

Although MEG provides exceptional temporal and cortical spatial resolution, it has limited sensitivity to deep subcortical structures such as the thalamus, striatum and brainstem nuclei. These regions are involved in arousal regulation and are implicated in LBD pathology. Integrating MEG with complementary modalities, including structural and functional MRI, or PET imaging could provide a more complete picture of the distributed neural circuits contributing to cognitive fluctuations.

The cross-sectional nature of our study precludes causal inferences about the progression of neural abnormalities over time. Longitudinal studies are needed to determine whether the identified dynamic brain state signatures and spectral biomarkers predict clinical trajectories, cognitive decline, or conversion from prodromal to syndromic stages. These future studies may also reveal whether MEG-based features can serve as early indicators of therapeutic response or emerging pathology, such as alpha-synuclein accumulation or dopaminergic deficits.

The relatively modest sample size, particularly within the LBD group, limits statistical power and generalizability. We mitigated these constraints in several ways. First, the HMM approach leverages the long, high-temporal-resolution MEG time series to estimate within-participant state sequences and summary metrics (e.g. fractional occupancy), reducing reliance on large between-group sample sizes for the primary state-dynamic estimates.^24^ Second, we used nonparametric permutation-based inference, which is well suited for small samples because it minimizes distributional assumptions.^78^ Third, learning the state structure from a large normative MEG cohort (CamCAN) improves estimation of state patterns and enhances model stability. Despite the modest cohort, we observed consistent patterns across participants and significant correlations with independent clinical measures, lending credibility to the findings. Importantly, recruiting clinically well-characterized LBD patients who can tolerate advanced MEG protocols is inherently challenging, and MEG itself remains a relatively rare technology. Within this context, our study represents one of the first LBD cohorts examined with MEG. Even so, larger, multicentre studies will be crucial to replicate and extend these results.

A further limitation is the absence of an AD dementia control group. Because LBD is frequently misdiagnosed as AD in clinical settings, future studies should include adequately powered AD and other dementia cohorts to directly test the diagnostic specificity of state-dynamic MEG markers.

HMM model order *K* must be specified as a priori, and alternative *K* values could yield finer subdivisions of network configurations. We used *K* = 6 as a parsimonious, commonly adopted resolution in resting-state MEG-HMM and mitigated local-minima risk via multiple initializations and selection of the lowest-free-energy solution. Future studies with larger clinical cohorts can more formally evaluate *K*-dependence and generalization using reproducibility- or cross-validated criteria.

Medication exposure can influence both oscillatory activity and cognitive fluctuations and should therefore be considered when interpreting group differences. Dopaminergic therapies are known to modulate cortical oscillations—particularly beta-band activity—in parkinsonian disorders, while cholinesterase inhibitors can alter spectral profiles by reducing slow-frequency power and enhancing faster rhythms.^79-81^ In the present cohort, medication use reflected routine clinical care: participants with LBD were treated with levodopa–carbidopa in combination with donepezil, whereas participants with Parkinson’s disease without dementia received levodopa–carbidopa only. Importantly, no participants were taking dopamine agonists or sedating psychoactive medications (e.g. antipsychotics or benzodiazepines) that are known to markedly affect EEG/MEG spectral properties.^82^ Although this relative homogeneity reduces major pharmacological confounding, future studies with larger samples will be required to more definitively disentangle disease-related effects from medication-specific influences, ideally through detailed covariate modelling and/or controlled on–off medication paradigms.

Resting-state MEG, particularly during eyes-closed acquisition, is sensitive to fluctuations in vigilance and arousal, which can influence spectral estimates.^83^ Although procedural steps were taken to reduce drowsiness-related effects, formal vigilance scoring was not performed, and residual arousal variability cannot be fully excluded. Future studies should incorporate standardized vigilance annotation (manual or automated) and larger samples to more definitively disentangle disease-related network dynamics from arousal-related variability.

Finally, our analysis was restricted to resting-state data, which, while informative, does not fully capture how cognitive fluctuations manifest during active task engagement. Future studies incorporating task-based paradigms, especially those targeting attention, executive function, or visual processing, may reveal how state-dependent dynamics interact with external cognitive demands and uncover neural mechanisms that predict vulnerability to cognitive lapses during behaviour.

## Conclusion

This study provides compelling evidence that LBD is characterized by distinct alterations in brain state dynamics and widespread spectral slowing, both of which closely correspond to the severity of cognitive fluctuations, a core clinical feature of the disease. Leveraging the high temporal resolution of MEG and advanced HMM, we identified neurophysiological signatures that distinguish LBD from PD without dementia and cognitively NC. These signatures, including aberrant state occupancy, elevated TBRs and region-specific correlations with cognitive fluctuation severity, represent promising candidate biomarkers for diagnosis, disease monitoring and potentially guiding therapeutic strategies. By advancing our mechanistic understanding of the dynamic neural processes underlying fluctuating cognition in LBD, this work lays the foundation for developing sensitive and objective tools to support early detection, track disease progression and assess treatment efficacy.

## Supplementary Material

fcag236_Supplementary_Data

## Data Availability

The preprocessing scripts, hidden Markov model (HMM) analysis code and visualization pipelines used in this study are openly available in a dedicated GitHub repository: https://github.com/sadeqi60/MEG_HMM_LBD.
