# Supplementary material for: Dynamic, state-dependent characteristics of cognitive fluctuations in Lewy body dementia: a magnetoencephalography study
Source: Brain Commun. 2026 Jun 24;8(4):fcag236. doi: 10.1093/braincomms/fcag236 (PMC13378770; doi:10.1093/braincomms/fcag236)
Supplement: fcag236_Supplementary_Data [file fcag236_supplementary_data.docx]

**Supplementary Materials**

## Group Differences in Brain State Occupancy

In this study, dementia with Lewy bodies (DLB) and Parkinson’s disease dementia (PDD) were grouped under Lewy body dementia (LBD) for primary analyses, consistent with their shared Lewy body pathology and overlapping clinical features. The LBD cohort comprised DLB (n = 5) and PDD (n = 2). Because the PDD subgroup is very small, formal inferential comparisons between DLB and PDD (or modeling diagnostic subtype as a covariate) would be underpowered and potentially unstable. To improve interpretability without overstating inference, we provide descriptive subgroup summaries for state dynamics and spectral features (fractional occupancy [FO], state-resolved power spectral density [PSD], and theta/beta ratio [TBR]). These subgroup displays are intended to help readers assess whether patterns appear qualitatively similar across LBD subtypes and to motivate future, adequately powered studies.

**Supplementary Figure 1** illustrates differences in brain state dynamics between DLB and PDD using FO derived from hidden Markov modeling (HMM). Both DLB and PDD show a broadly similar pattern, characterized by significantly increased occupancy of State 2 and reduced occupancy of State 6 relative to cognitively normal controls and Parkinson’s disease without dementia, supporting the view that they share core Lewy body–related network alterations.


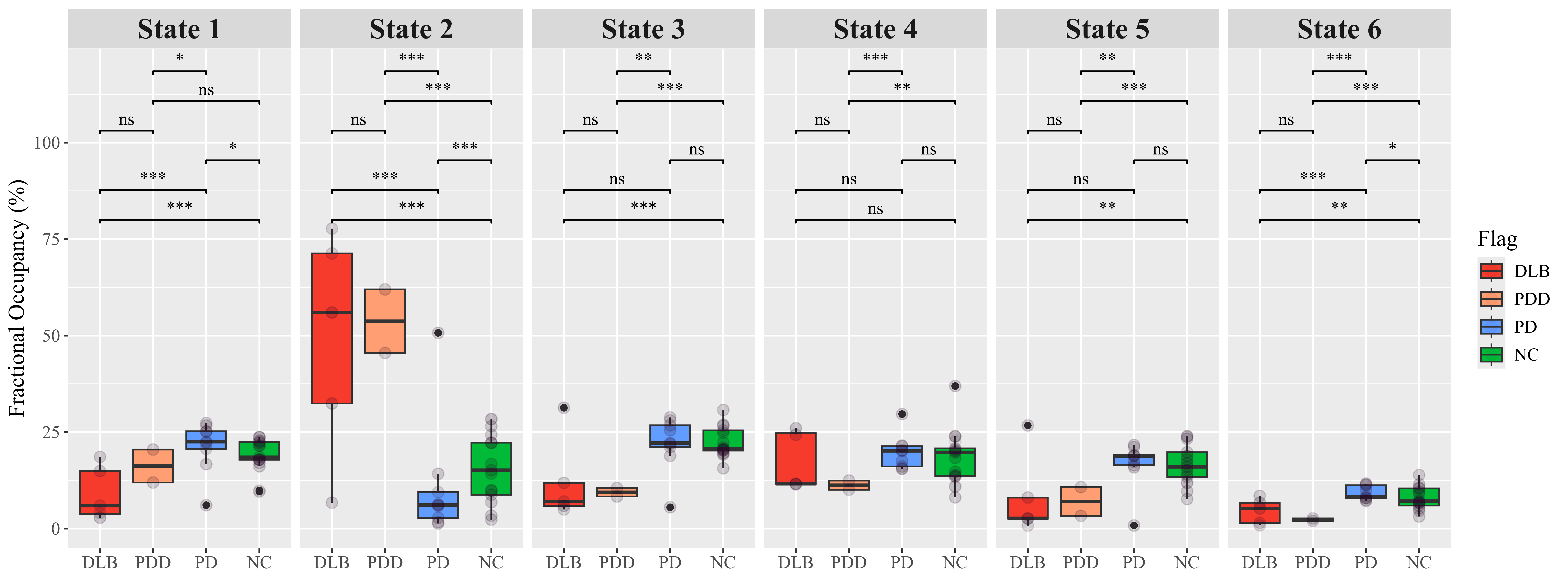


**Supplementary Figure 1. Brain state dynamics in Lewy body–related dementias revealed by hidden Markov modeling.** Boxplots depict the fractional occupancy (FO) of the six recurrent brain states derived from time-delay embedded hidden Markov modeling (TDE-HMM) across four groups: dementia with Lewy bodies (DLB, n=5 participants), Parkinson’s disease dementia (PDD, n=2 participants), Parkinson’s disease without dementia (PD, n=9 participants), and cognitively normal controls (NC, n=15 participants). The experimental unit is the individual participant, and each datapoint represents one independent subject’s mean fractional occupancy across the five sessions for a given state. Group differences were assessed using nonparametric permutation t-tests (5,000 label permutations) with Bonferroni correction for multiple states. Asterisks denote significant between-group differences (**P* < 0.05, ***P* < 0.01, ****P* < 0.001; ‘n.s.’: not significant). Both DLB and PDD subgroups show qualitatively elevated FO in State 2 and reduced FO in State 6 relative to NC and PD groups. No formal statistical comparison was performed between DLB and PDD due to the very small PDD sample size (n=2). PD participants showed the highest FO in State 1, while NC participants displayed a more balanced distribution with peaks in States 3 and 5.

## Spectral Slowing and Theta/Beta Power Ratios

**Supplementary Figure 2A** demonstrates that spectral slowing is more pronounced in both DLB and PDD compared with PD and cognitively normal controls. Although the overall spectral profiles of DLB and PDD appear broadly similar, direct statistical comparison between the two groups was not performed due to the limited sample sizes (n = 5 for DLB and n = 2 for PDD).

Regional analysis of TBR revealed distinct but overlapping spatial patterns of spectral slowing in DLB and PDD, predominantly within State 2 (**Supplementary Figure 2B**). In DLB, the highest TBR was observed in the left anterior cingulate and medial prefrontal cortex (TBR = 11.31), followed by strong involvement of auditory regions, including the right early auditory cortex (TBR = 10.62) and right auditory association cortex (TBR = 9.93). Additional elevated TBRs were evident in multimodal and control-related regions such as the insular and frontoparietal operculum, inferior parietal cortex within the task-negative network, premotor cortex, and the temporal–parieto–occipital junction, indicating widespread slowing across frontal, parietal, and auditory networks. In contrast, PDD showed its peak TBR in the right auditory association cortex (TBR = 12.44), exceeding values observed in DLB, with similarly high TBRs in the right and left early auditory cortices and the left temporal–parieto–occipital junction. PDD additionally demonstrated prominent involvement of posterior regions, including the posterior cingulate cortex and multiple visual areas (primary visual cortex, ventral stream, and MT+ complex), suggesting a stronger posterior and sensory-dominant profile of spectral slowing. Overall, while both DLB and PDD share a common State 2–centered pattern with marked auditory and associative cortex involvement, DLB appears to show relatively greater frontal–cingulate and control-network engagement, whereas PDD is characterized by more pronounced auditory and posterior cortical slowing.

To quantify the magnitude of group differences in spectral power beyond statistical significance, effect sizes were estimated using Hedges’ *g*, which provides a bias-corrected measure suitable for unequal and modest sample sizes. Group comparisons were performed after adjusting for sex, age, and years of education as covariates to account for potential demographic confounds known to influence spectral power. Reporting adjusted effect sizes allows direct comparison of the strength and spatial distribution of disease-related power alterations across groups, independent of these covariates, and complements the inferential results by emphasizing biologically meaningful differences.

(A)


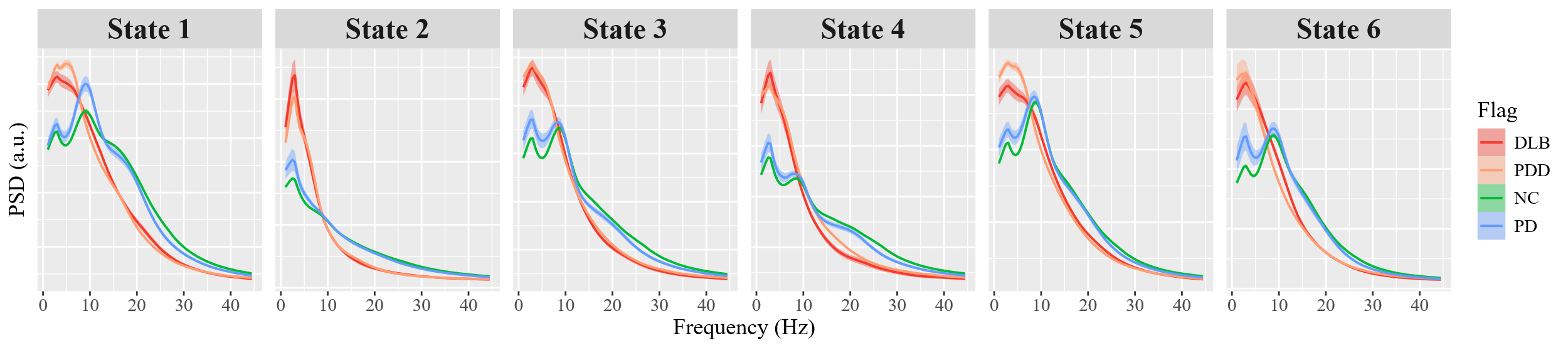


(B)


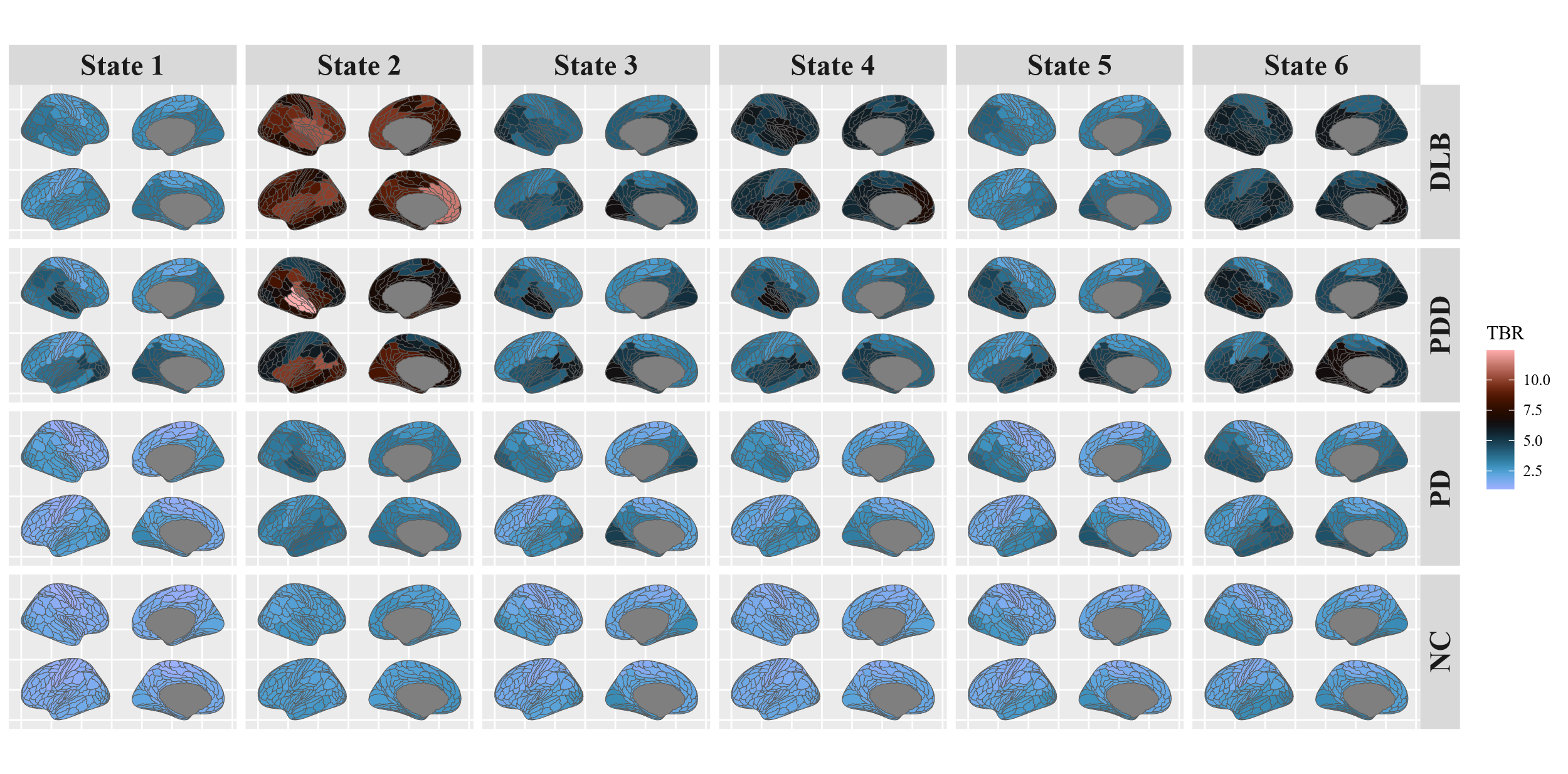


**Supplementary Figure 2. Spectral slowing and spatial distribution of theta–beta ratios across brain states in Lewy body–related dementias.** **(A)** Power spectral density (PSD) profiles averaged across participants showing frequency-dependent power across the six brain states in dementia with Lewy bodies (DLB, n=5), Parkinson’s disease dementia (PDD, n=2), Parkinson’s disease without dementia (PD, n=9), and cognitively normal controls (NC, n=15). The experimental unit is the individual participant. **(B)** Cortical surface maps (Glasser 52 atlas) display the distribution of mean theta–beta power ratios (TBR) across the six brain states for each group. No formal inferential statistics were performed between DLB and PDD due to the small PDD sample size (n=2). Both DLB and PDD exhibit marked spectral slowing (elevated delta/theta and reduced alpha/beta power), most prominent in States 2 and 6. Highest regional TBR values in State 2 were observed in frontal-cingulate and auditory regions for DLB and in auditory and posterior regions for PDD.

**Supplementary Figures 3–6** present adjusted group differences in the TBR across brain states to evaluate disease-specific patterns of spectral slowing. For each comparison, adjusted differences were estimated after controlling for sex, age, and years of education. To assess the robustness of these findings, parallel sensitivity analyses were performed that additionally included global TBR as a covariate. Effect sizes for statistically significant adjusted differences are reported using Hedges’ *g*, enabling comparison of the magnitude of group effects across states and contrasts while accounting for sample size bias. Together, these analyses demonstrate the stability and relative strength of state-dependent TBR alterations across diagnostic groups.

(A)


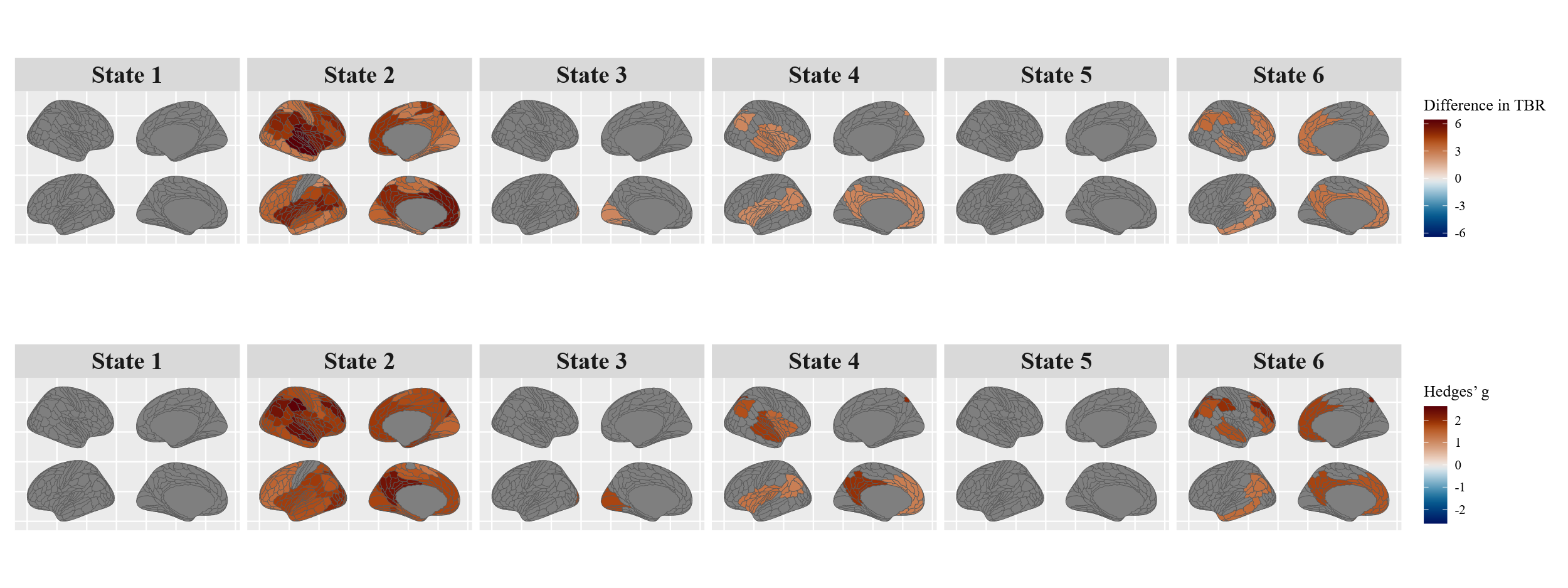


(B)


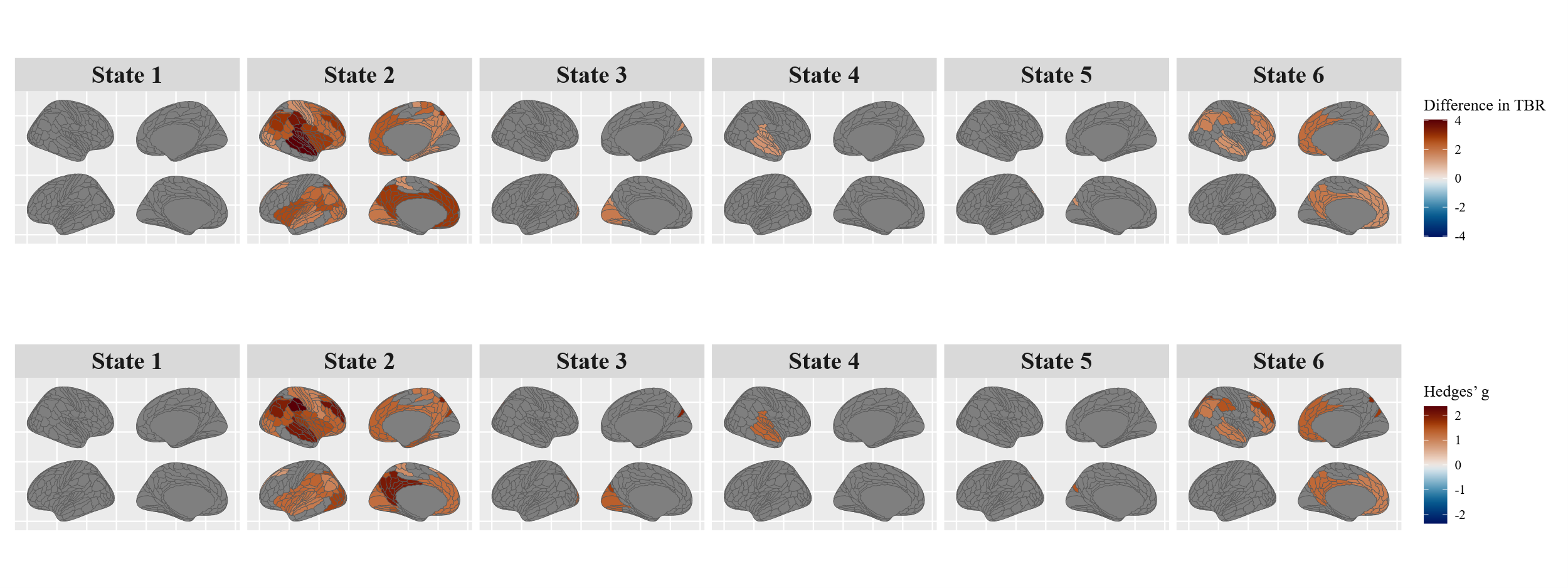


**Supplementary Figure 3. Adjusted theta/beta ratio (TBR) differences between NC and LBD across brain states.** **(A)** Adjusted differences in TBR between cognitively normal controls (NC, n=15) and Lewy body dementia (LBD, n=7) across the six brain states after controlling for sex, age, and years of education.
**(B)** Sensitivity analysis showing adjusted TBR differences with additional adjustment for global TBR. The experimental unit is the individual participant. Analyses used permutation-based general linear models (5,000 label shuffles) with FDR correction. Hedges’ g effect sizes are reported for brain states with statistically significant adjusted differences (FDR-corrected *P* < 0.05). States 2 and 6 showed the largest LBD-related increases in TBR.

(A)


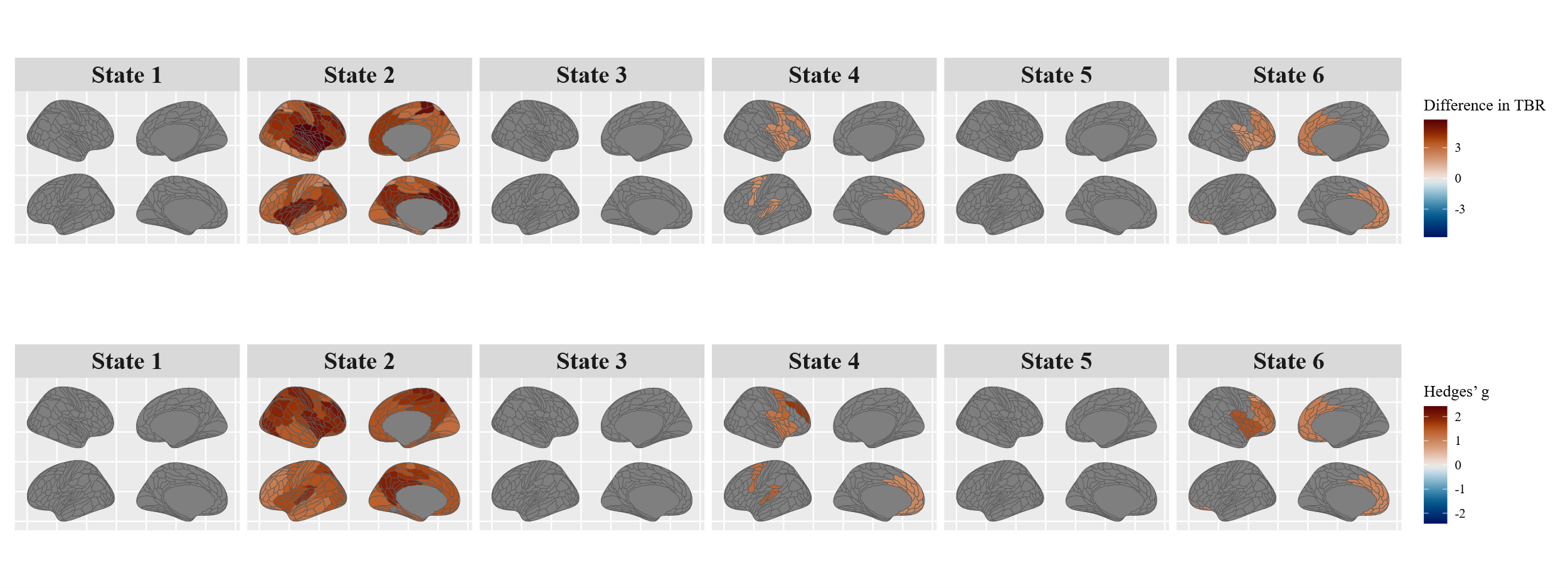


(B)


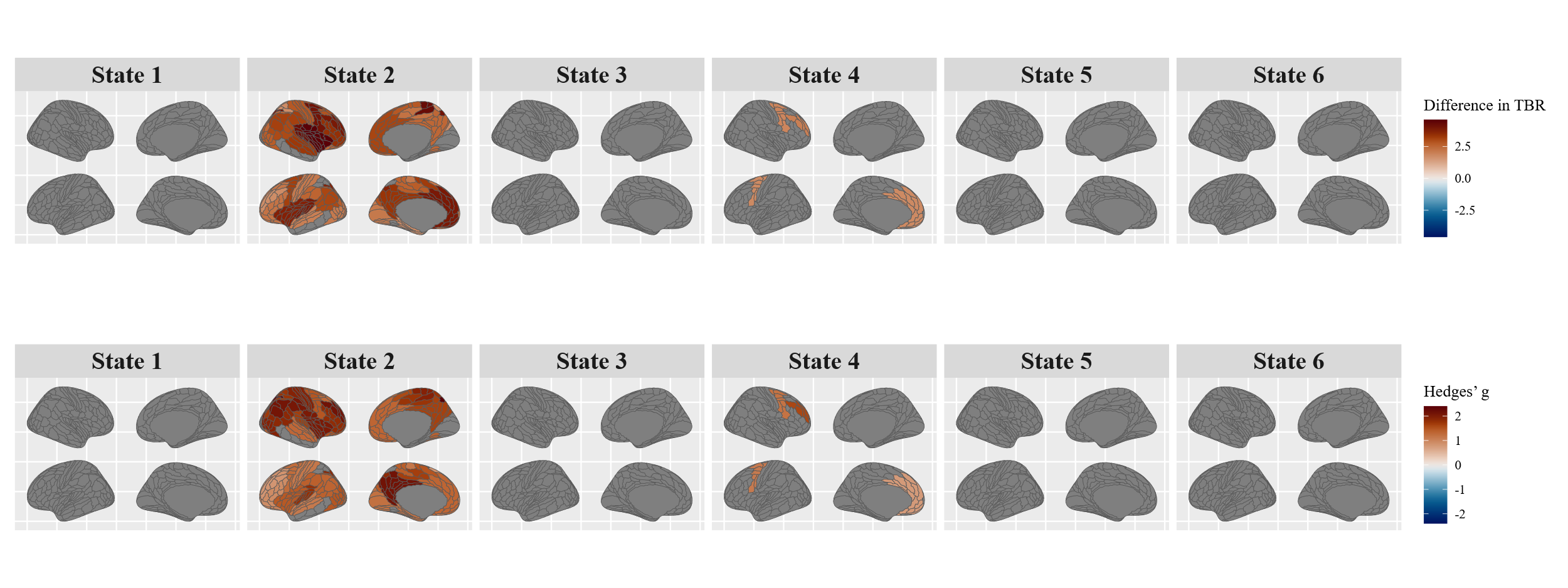


**Supplementary Figure 4. Adjusted theta/beta ratio (TBR) differences between PD and LBD across brain states.** **(A)** Adjusted differences in TBR between Parkinson’s disease without dementia (PD, n=9) and Lewy body dementia (LBD, n=7) across the six brain states after controlling for sex, age, and years of education. **(B)** Sensitivity analysis including global TBR as an additional covariate. The experimental unit is the individual participant. Permutation-based general linear models (5,000 label shuffles) were used with FDR correction. Hedges’ g values are provided for statistically significant adjusted differences (FDR-corrected *P* < 0.05).

(A)


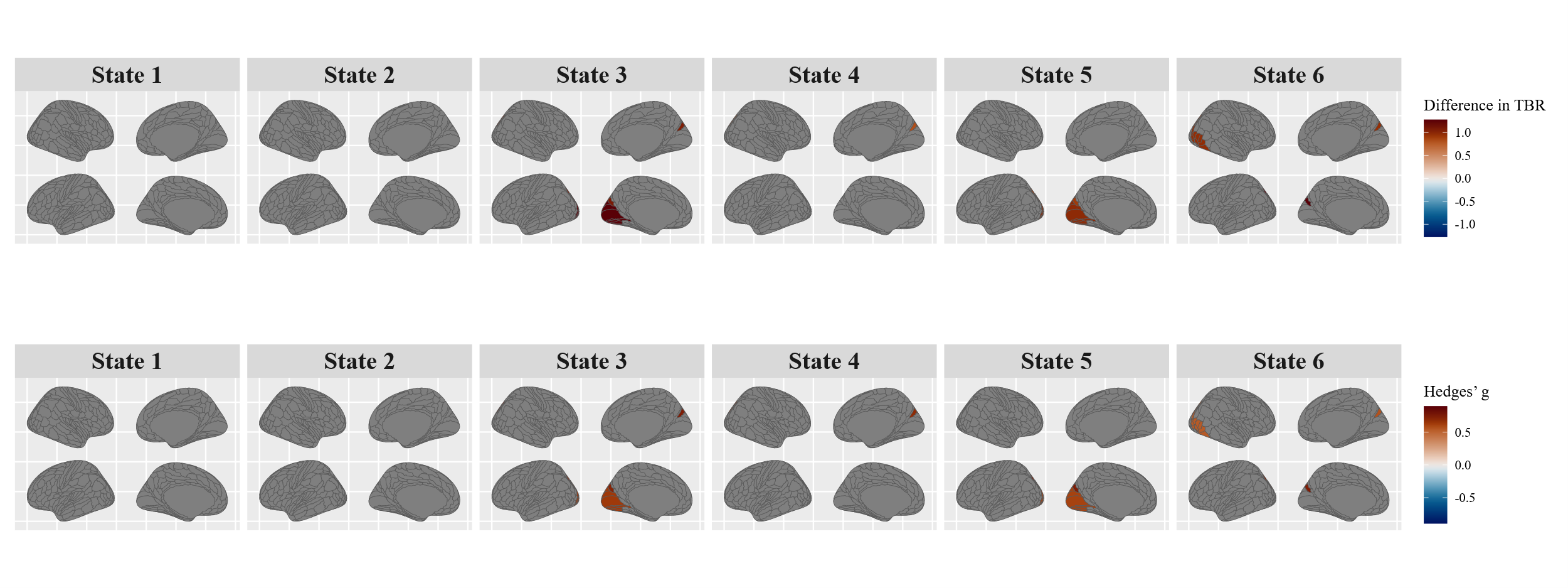


(B)


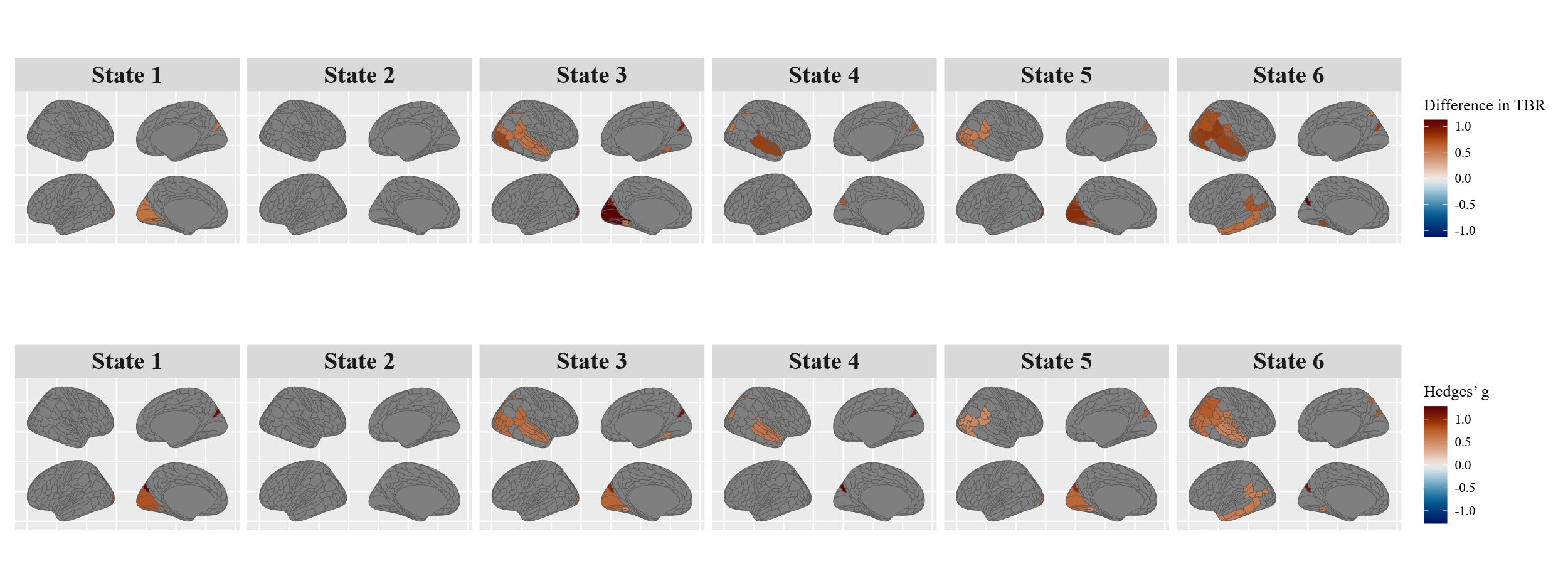


**Supplementary Figure 5. Adjusted theta/beta ratio (TBR) differences between PD and NC across brain states.** **(A)** Adjusted differences in TBR between Parkinson’s disease without dementia (PD, n=9) and cognitively normal controls (NC, n=15) across the six brain states after controlling for sex, age, and years of education. **(B)** Sensitivity analysis with additional adjustment for global TBR. The experimental unit is the individual participant. Analyses were performed using permutation-based general linear models (5,000 label shuffles) with FDR correction. Hedges’ g effect sizes are reported for significant adjusted differences (FDR-corrected *P* < 0.05).

(A)


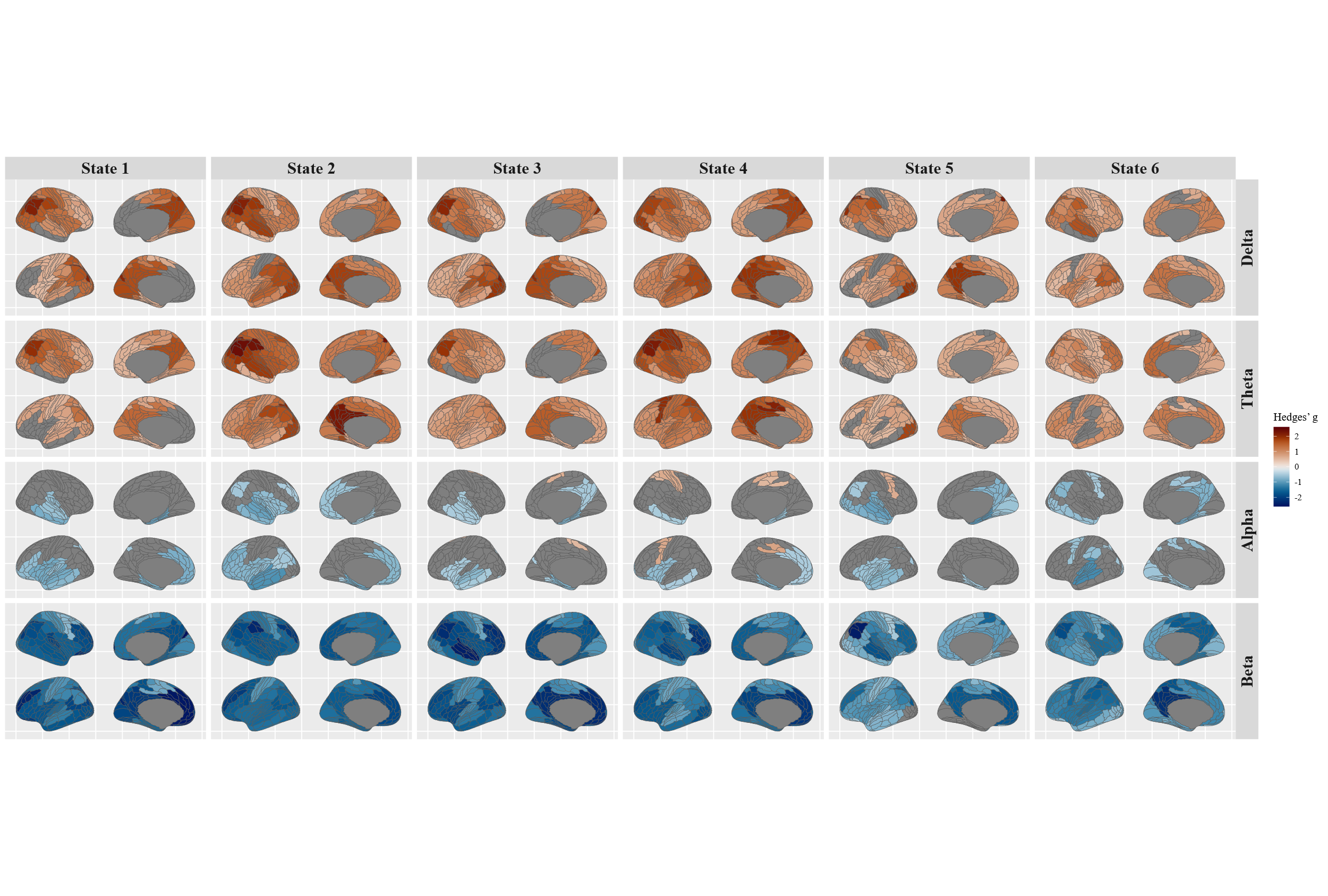


(B)


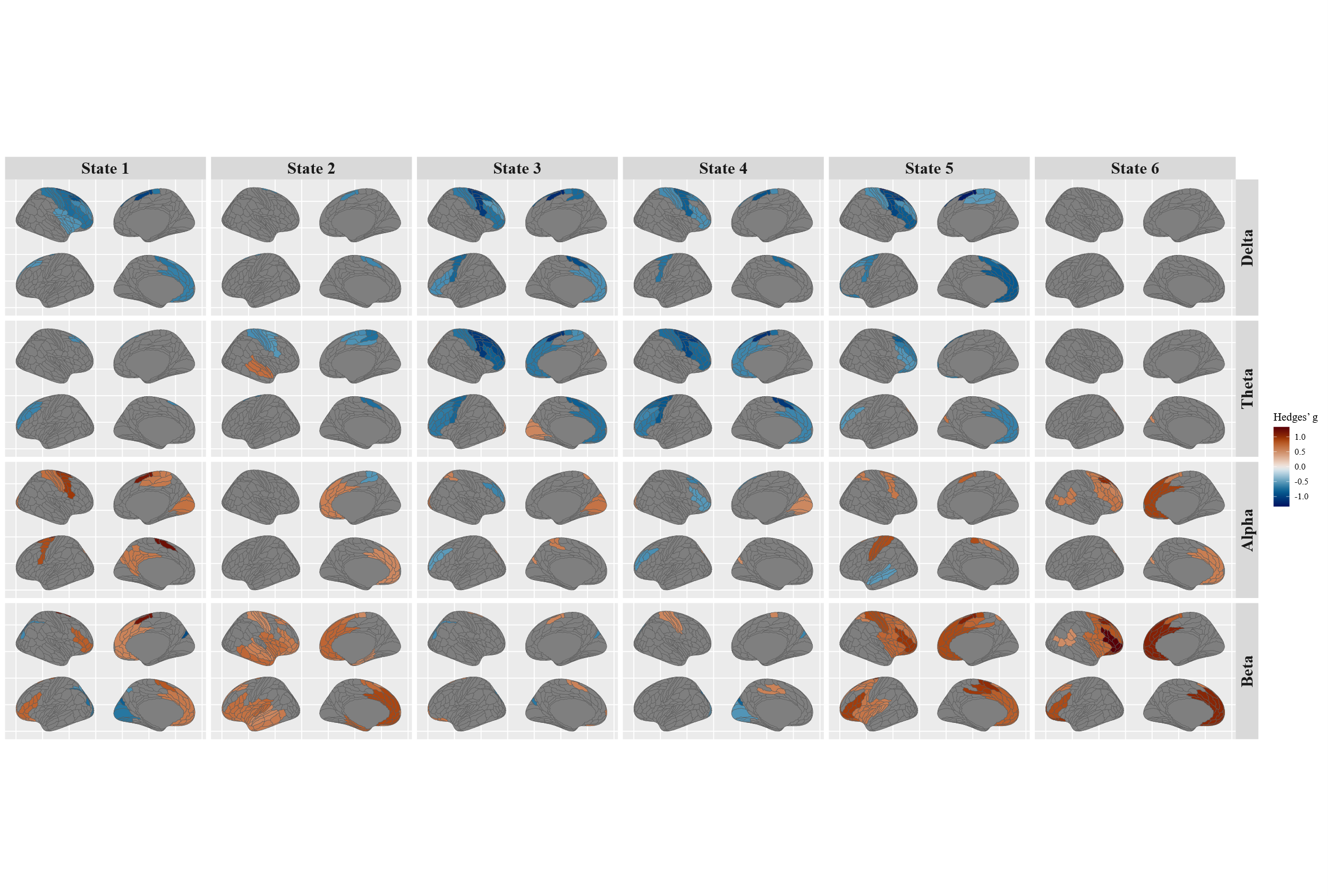


**Supplementary Figure 6. Effect sizes of adjusted group differences in spectral power.** **(A)** Cortical surface maps showing Hedges’ g for adjusted spectral power differences between Lewy body dementia (LBD, n=7) and cognitively normal controls (NC, n=15). **(B)** Cortical surface maps showing Hedges’ g for adjusted spectral power differences between Parkinson’s disease without dementia (PD, n=9) and NC (n=15). All effect sizes were derived from permutation-based general linear models adjusted for sex, age, and years of education (5,000 label shuffles, FDR-corrected *P* < 0.05). The experimental unit is the individual participant. Only regions with statistically significant adjusted differences are displayed. Positive values indicate higher power in the patient group; negative values indicate lower power.

The standard error (SE) derived from jackknife resampling provides a direct measure of the stability and reliability of ROI-level effect estimates across subjects (**Supplementary Figure 7**). Unlike point estimates of correlation or power that can be disproportionately influenced by individual participants, the jackknife SE quantifies how much an effect varies when each subject is systematically omitted. Small SE values indicate that the observed association is robust and not driven by outliers, whereas large SE values suggest sensitivity to subject-level variability. In the context of LBD, where inter-individual heterogeneity is pronounced, incorporating jackknife SE ensures that reported state- and frequency-specific effects reflect consistent neurophysiological patterns rather than sample-specific noise.


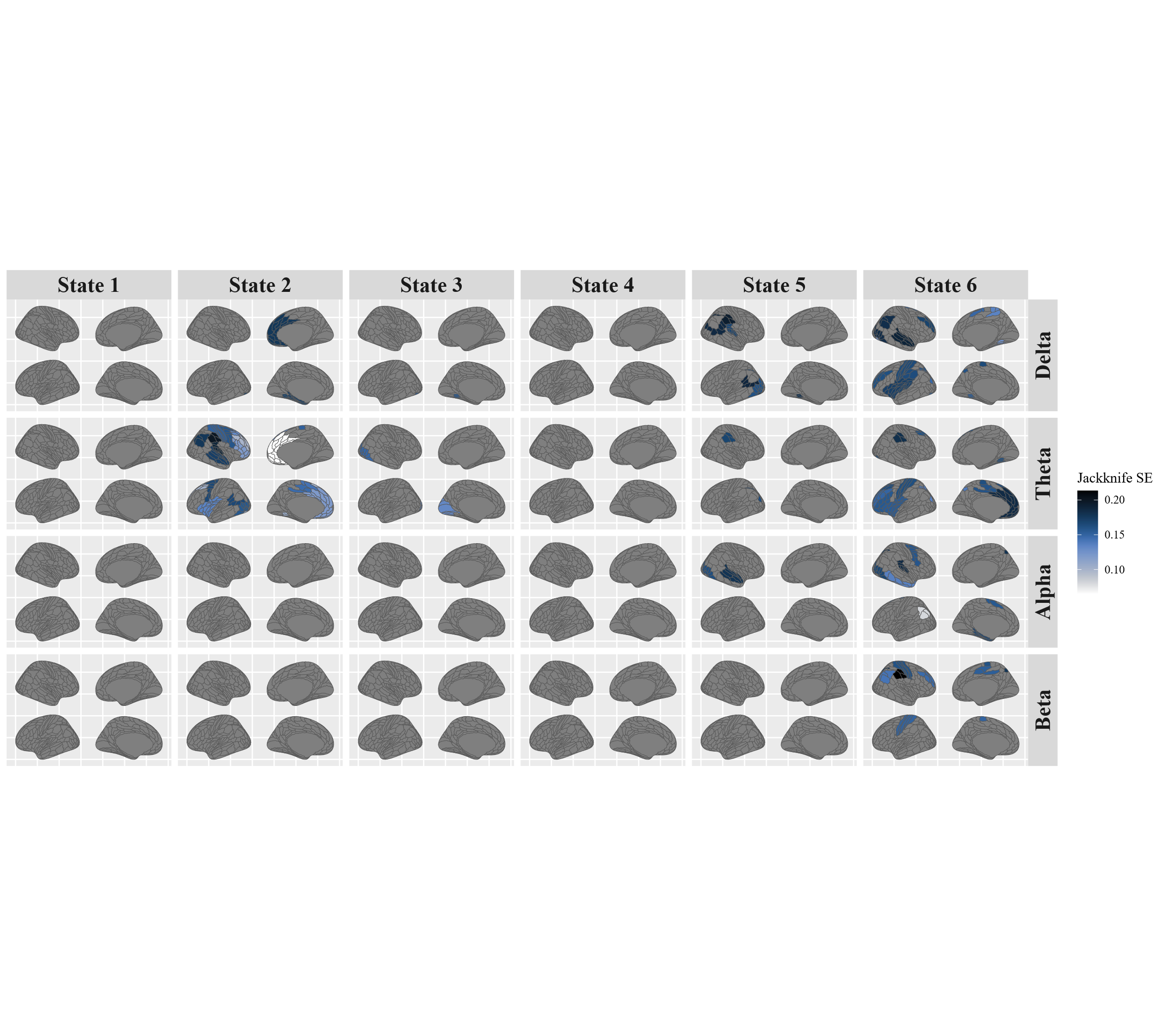


**Supplementary Figure 7. Jackknife-derived standard error supporting robustness of CAF–spectral power correlations.** The figure shows the jackknife-derived standard error (SE) of ROI-level Spearman correlations between Clinician Assessment of Fluctuation (CAF) scores and regional spectral power across the six brain states and frequency bands in the LBD group (n=7 participants). The experimental unit is the individual participant. Jackknife SE was estimated using leave-one-subject-out resampling. Correlations were considered robust if they met all three criteria: FDR-corrected *P* < 0.05 (within state–frequency band), 100% sign consistency across jackknife iterations, and absolute correlation magnitude exceeding the jackknife SE (|ρ| > SE). Lower SE values indicate higher reliability of the observed state- and frequency-specific associations with cognitive fluctuations. This supplementary analysis supports the primary CAF–spectral power correlations shown in main **Figure 4**.

1. ***Association Between Spectral Power and Cognitive Fluctuations: MFS and ODF***

Analysis of Mayo Fluctuation Score (MFS) revealed complementary patterns of spectral power across states and regions. Higher MFS was consistently associated with reductions in alpha-band power, most prominently in State 6 across posterior, parietal, and temporal regions, including the right temporal–parieto–occipital junction and left inferior parietal task-negative network, reflecting widespread alpha suppression with increased cognitive fluctuation. In parallel, positive correlations were observed primarily in low-frequency bands, with theta and delta power in visual, parietal, and auditory cortices showing strong state-specific effects, particularly in States 5 and 6—for example, theta power in the right dorsal stream visual cortex (State 5) and delta power in the left posterior cingulate (State 6). Together, these findings indicate that higher cognitive fluctuation is linked to a dual pattern of decreased alpha activity alongside enhanced low-frequency power in posterior and multimodal networks, highlighting coordinated alterations in large-scale network dynamics underlying fluctuating cognition (**Supplementary Figure 8**).

Analysis of One Day Fluctuation Assessment (ODF) scale correlations revealed a pronounced dual pattern of state- and frequency-specific effects across cortical regions. Negative associations were strongest in alpha-band power, particularly in State 6, encompassing the auditory association cortex, lateral temporal cortices, inferior parietal task-negative network, superior medial parietal cortex, and dorsal and ventral visual areas, reflecting widespread alpha suppression with higher ODF. In contrast, positive correlations were observed primarily in delta and theta bands, involving motor, premotor, intraparietal, dorsolateral prefrontal, and visual cortices across States 2, 3, 5, and 6, indicating enhanced low-frequency activity with increasing ODF. Together, these results suggest that higher ODF is associated with reduced alpha activity alongside elevated delta and theta power in posterior, parietal, and multimodal networks, highlighting coordinated large-scale network modulation related to fluctuating cognitive states (**Supplementary Figure 9**).


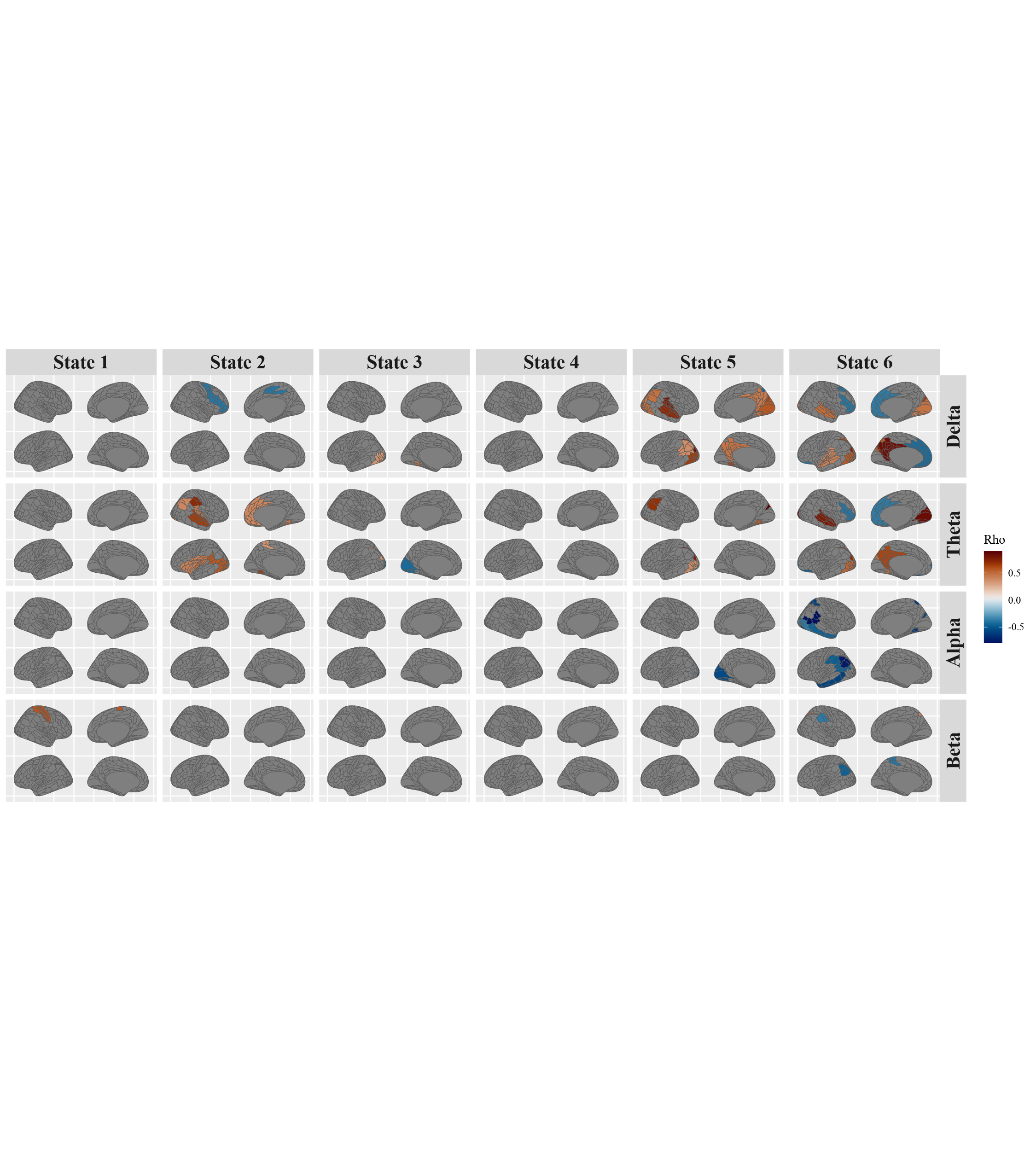


**Supplementary Figure 8. Neural correlates of cognitive fluctuations measured by the Mayo Fluctuations Scale (MFS) in LBD.** Cortical surface maps show Spearman correlations (ρ) between MFS scores and regional spectral power across frequency bands and the six brain states in the LBD group (n=7 participants). The experimental unit is the individual participant. Red colors indicate positive correlations; blue colors indicate negative correlations. Only statistically significant associations are displayed (permutation-based Spearman correlations, 5,000 permutations, with FDR correction within each state–frequency band, *P* < 0.05). Key examples include positive θ-band correlation in the right primary and early visual cortex during State 6 (ρ = 0.83), positive δ-band correlation in the left posterior cingulate cortex during State 6 (ρ = 0.82), and negative α-band correlation in the right temporal-parieto-occipital junction during State 6 (ρ = −0.79). These MFS-based findings provide complementary fluctuation-scale associations to the primary CAF-based results shown in main **Figure 4**.


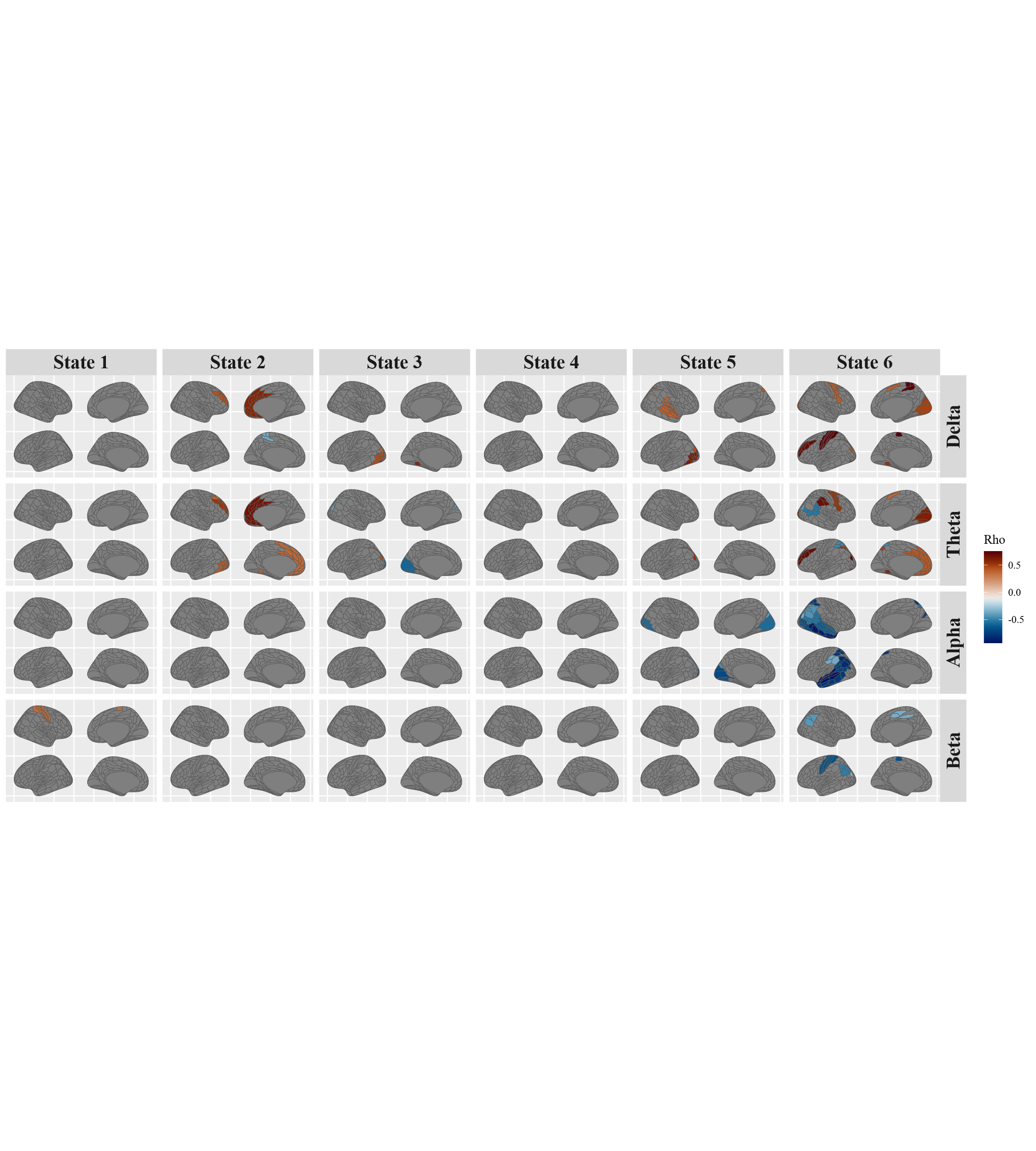


**Supplementary Figure 9. Neural correlates of cognitive fluctuations measured by the One Day Fluctuation Assessment Scale (ODF) in LBD.** Cortical surface maps show Spearman correlations (ρ) between ODF scores and regional spectral power across frequency bands and the six brain states in the LBD group (n=7 participants). The experimental unit is the individual participant. Red colors indicate positive correlations; blue colors indicate negative correlations. Only statistically significant associations are displayed (permutation-based Spearman correlations, 5,000 permutations, with FDR correction within each state–frequency band, *P* < 0.05). Key examples include positive δ-band correlation in the right inferior somatosensory and motor cortex during State 6 (ρ = 0.75), positive θ-band correlation in the left intraparietal sulcus & PGP during State 6 (ρ = 0.73), and negative α-band correlation in the left auditory association cortex during State 6 (ρ = −0.79). These ODF-based findings provide complementary fluctuation-scale associations to the primary CAF-based results shown in main **Figure 4**.
